# Supplementary material for: RTN3 Is a Novel Cold-Induced Protein and Mediates Neuroprotective Effects of RBM3
Source: Curr Biol. 2017 Mar 6;27(5):638–50. doi: 10.1016/j.cub.2017.01.047 (PMC5344685; doi:10.1016/j.cub.2017.01.047)
Supplement: Document S1. Supplemental Experimental Procedures, Figures S1–S5, and Tables S1–S6 [file mmc1.pdf]

Current Biology, Volume *27*

Supplemental Information

RTN3 Is a Novel Cold-Induced Protein and Mediates Neuroprotective Effects of RBM3

Amandine Bastide, Diego Peretti, John R.P. Knight, Stefano Grosso, Ruth V. Spriggs, Xavier Pichon, Thomas Sbarrato, Anne Roobol, Jo Roobol, Davide Vito, Martin Bushell, Tobias von der Haar, C. Mark Smales, Giovanna R. Mallucci, and Anne E. Willis

Figure S1

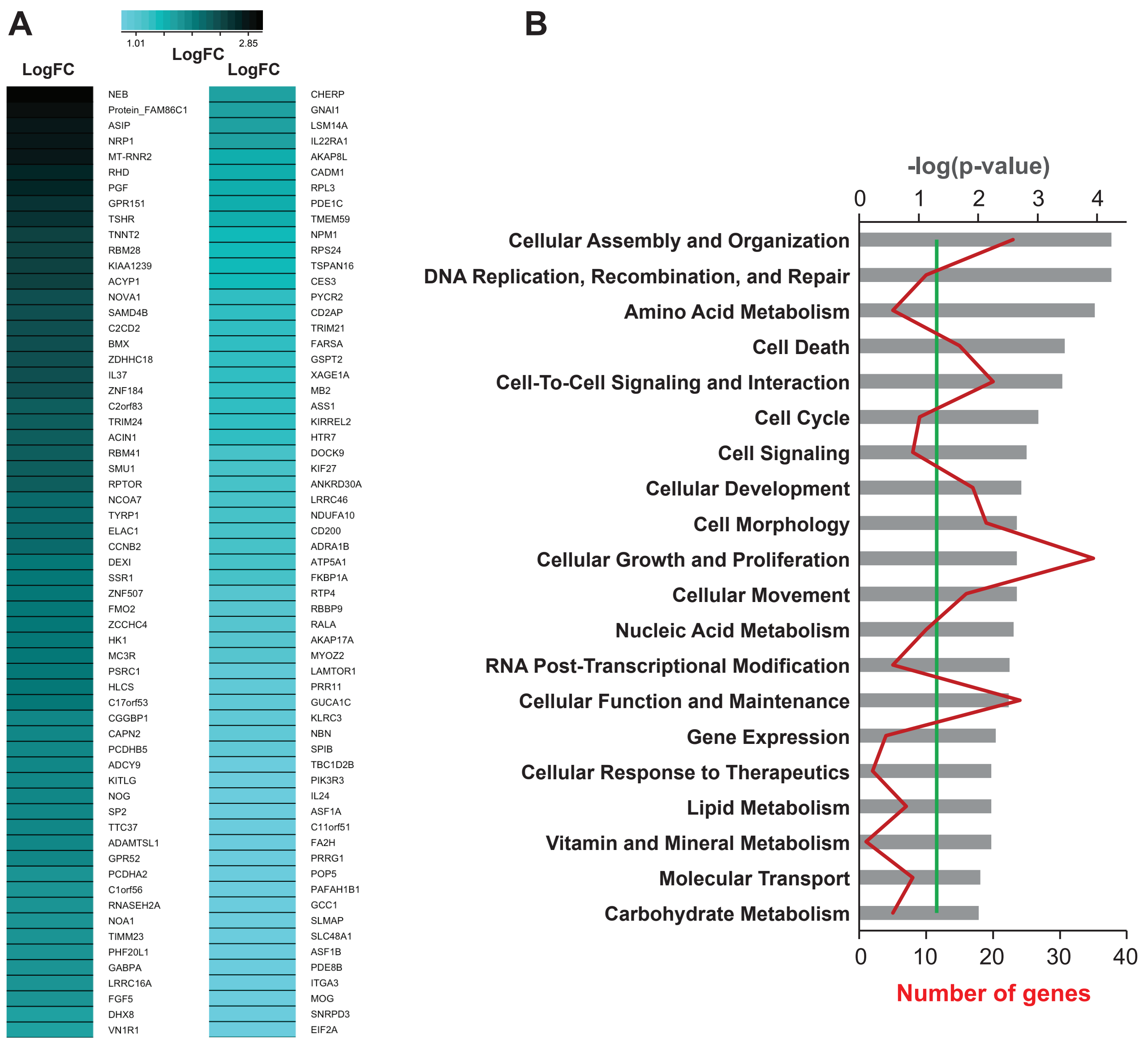
A
1.01
2.85
LogFC
LogFC
LogFC
NEB
Protein_FAM86C1
ASIP
NRP1
MT-RNR2
RHD
PGF
GPR151
TSHR
TNNT2
RBM28
KIAA1239
ACYP1
NOVA1
SAMD4B
C2CD2
BMX
ZDHHC18
IL37
ZNF184
C2orf83
TRIM24
ACIN1
RBM41
SMU1
RPTOR
NCOA7
TYRP1
ELAC1
CCNB2
DEXI
SSR1
ZNF507
FMO2
ZCCHC4
HK1
MC3R
PSRC1
HLCS
C17orf53
CGGBP1
CAPN2
PCDHB5
ADCY9
KITLG
NOG
SP2
TTC37
ADAMTSL1
GPR52
PCDHA2
C1orf56
RNASEH2A
NOA1
TIMM23
PHF20L1
GABPA
LRRC16A
FGF5
DHX8
VN1R1
CHERP
GNAI1
LSM14A
IL22RA1
AKAP8L
CADM1
RPL3
PDE1C
TMEM59
NPM1
RPS24
TSPAN16
CES3
PYCR2
CD2AP
TRIM21
FARSA
GSPT2
XAGE1A
MB2
ASS1
KIRREL2
HTR7
DOCK9
KIF27
ANKRD30A
LRRC46
NDUFA10
CD200
ADRA1B
ATP5A1
FKBP1A
RTP4
RBBP9
RALA
AKAP17A
MYOZ2
LAMTOR1
PRR11
GUCA1C
KLRC3
NBN
SPIB
TBC1D2B
PIK3R3
IL24
ASF1A
C11orf51
FA2H
PRRG1
POP5
PAFAH1B1
GCC1
SLMAP
SLC48A1
ASF1B
PDE8B
ITGA3
MOG
SNRPD3
EIF2A
B
-log(p-value)
0
1
2
3
4
Cellular Assembly and Organization
DNA Replication, Recombination, and Repair
Amino Acid Metabolism
Cell Death
Cell-To-Cell Signaling and Interaction
Cell Cycle
Cell Signaling
Cellular Development
Cell Morphology
Cellular Growth and Proliferation
Cellular Movement
Nucleic Acid Metabolism
RNA Post-Transcriptional Modification
Cellular Function and Maintenance
Gene Expression
Cellular Response to Therapeutics
Lipid Metabolism
Vitamin and Mineral Metabolism
Molecular Transport
Carbohydrate Metabolism
0
10
20
30
40
Number of genes

Figure S2

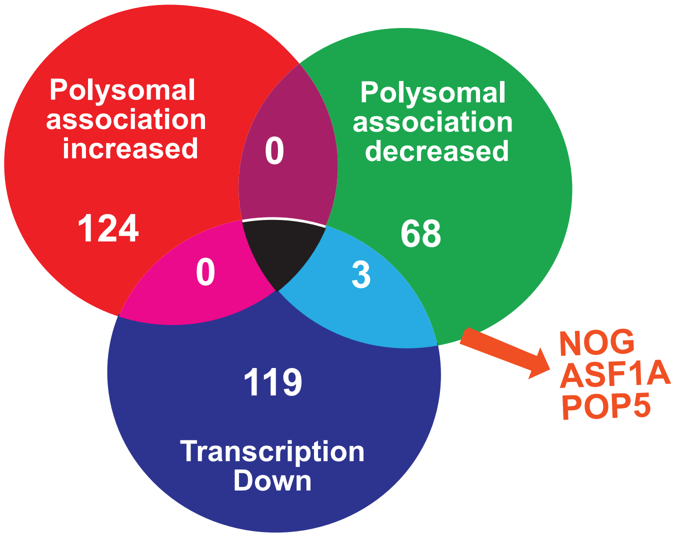

Polysomal association increased
124
0
Polysomal association decreased
68
0
3
119
Transcription Down
NOG
ASF1A
POP5

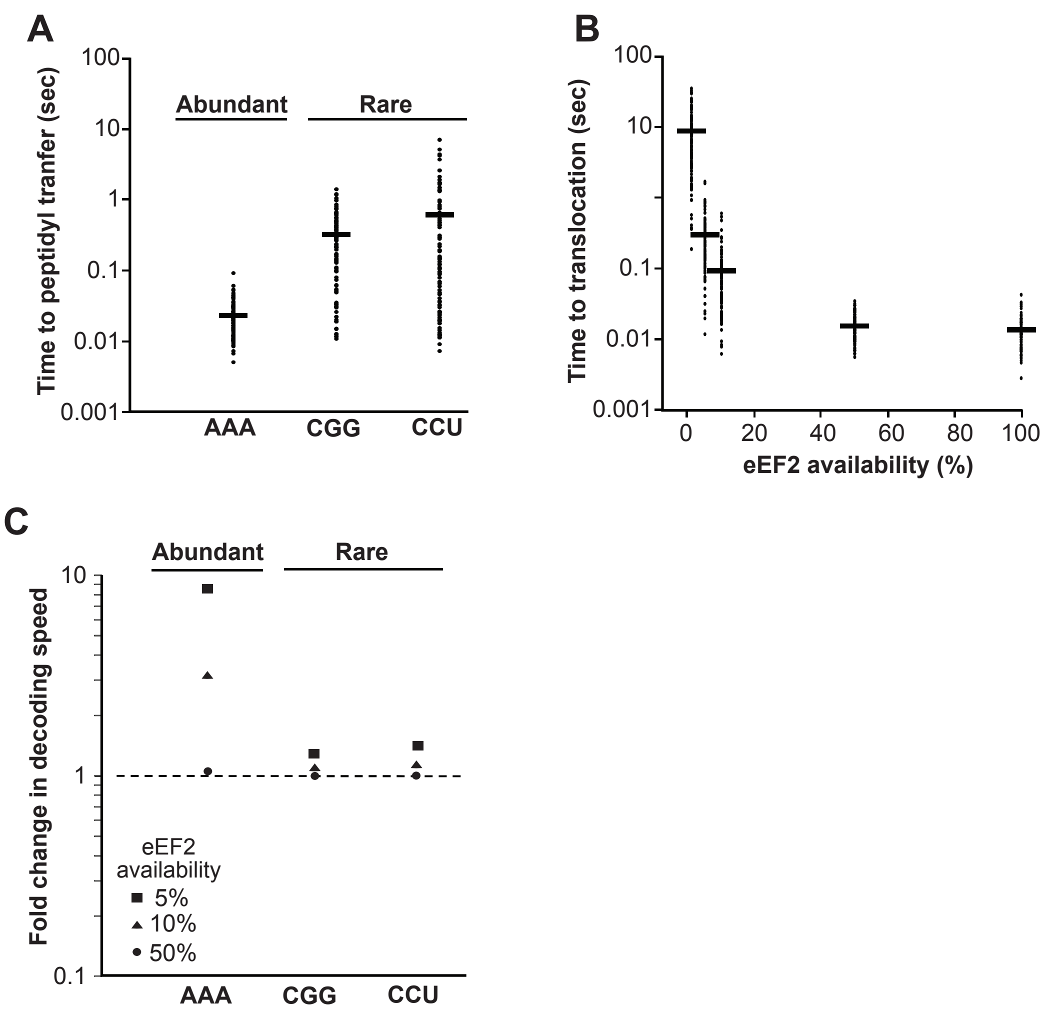
A
Time to peptidyl tranfer (sec)
100
10
1
0.1
0.01
0.001
Abundant
Rare
AAA
CGG
CCU
B
Time to translocation (sec)
100
10
0.1
0.01
0.001
0
20
40
60
80
100
eEF2 availability (%)
C
Fold change in decoding speed
10
1
0.1
Abundant
Rare
eEF2
availability
5%
10%
50%
AAA
CGG
CCU

A

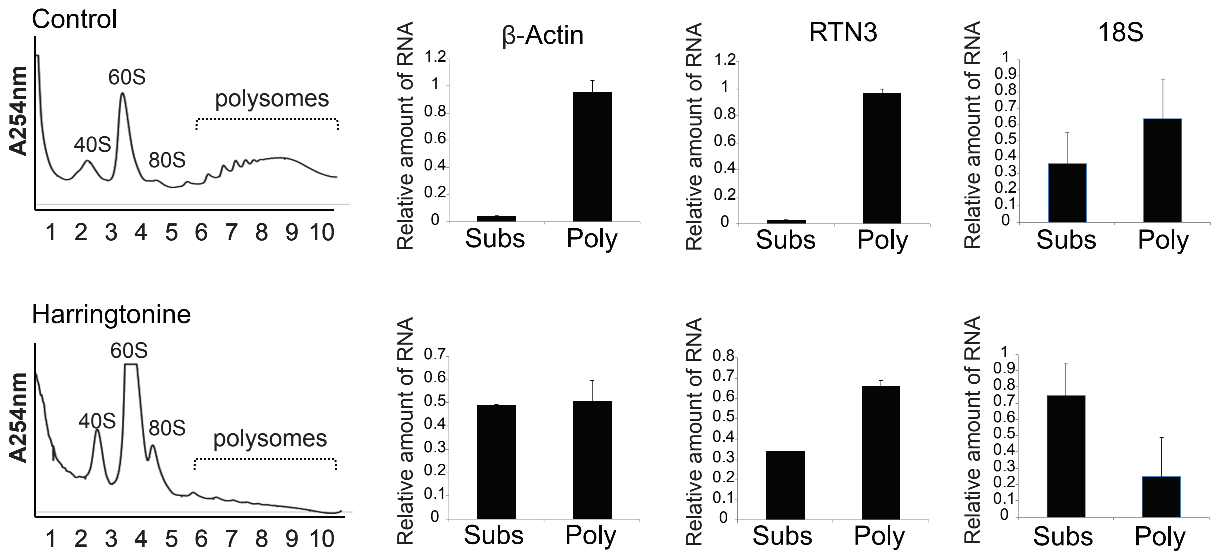
Control
A254nm
60S
40S
80S
polysomes
1 2 3 4 5 6 7 8 9 10
β-Actin
RTN3
18S
Relative amount of RNA
Subs
Poly
Harringtonine
A254nm
60S
40S
80S
polysomes
1 2 3 4 5 6 7 8 9 10
Relative amount of RNA
Subs
Poly

B

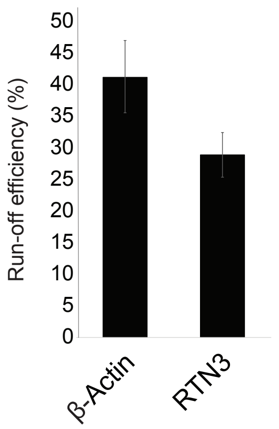
Run-off efficiency (%)
50
45
40
35
30
25
20
15
10
5
0
β-Actin
RTN3

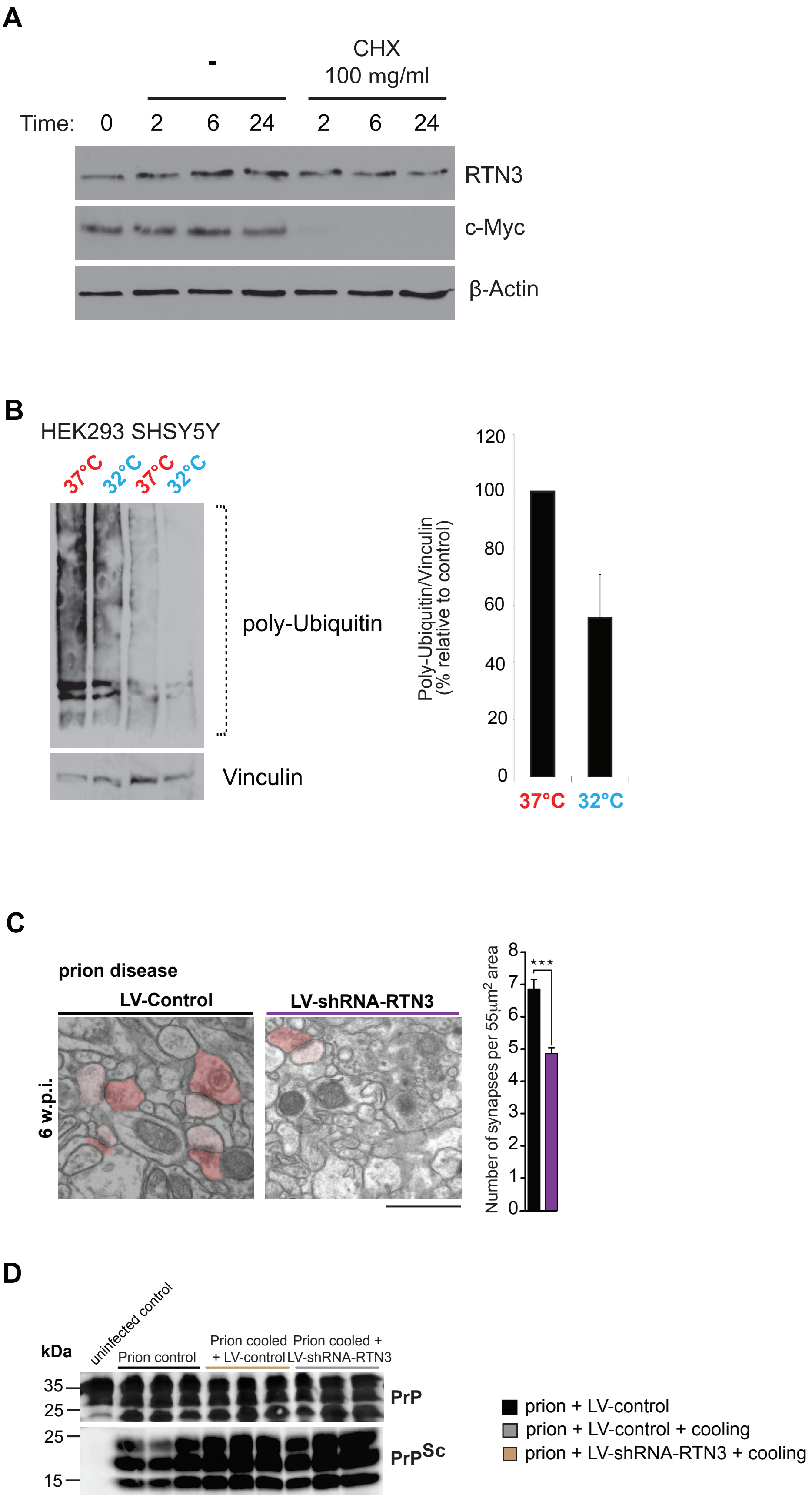
A
-
CHX
100 mg/ml
Time: 0 2 6 24 2 6 24
RTN3
c-Myc
β-Actin
B
HEK293 SHSY5Y
37°C 32°C 37°C 32°C
poly-Ubiquitin
Vinculin
Poly-Ubiquitin/Vinculin
(% relative to control)
120
100
80
60
40
20
0
37°C 32°C
C
prion disease
LV-Control
LV-shRNA-RTN3
6 w.p.i.
Number of synapses per 55µm² area
8
7
6
5
4
3
2
1
0
***
D
kDa
uninfected control
Prion control
Prion cooled + LV-control
Prion cooled + LV-shRNA-RTN3
35
25
25
15
PrP
PrPSc
prion + LV-control
prion + LV-control + cooling
prion + LV-shRNA-RTN3 + cooling

Figure S1. Transcriptional profiling following mild coldshock. Related to Figure 1

(A) Genes shown are those with a 2-fold change and a statistically significant difference between control and cooled conditions. Values represent the mean, normalised log_2 ratio of transcript level in cold-shock versus control conditions.

(B) Functional classification of genes down-regulated at transcriptional level following cold shock. Biological functions associated with regulated genes, obtained from the Ingenuity Pathways Analysis. p-value (threshold $p < 0.05$) for each biological function are represented by the grey bar chart, number of genes per category is represented by the purple line.

Figure S2. Venn diagram of to show genome-wide changes on cold shock. Related to Figure 1, Figure S1 and Tables S1-S3

The Venn diagram shows the number of overlapping genes between polysomal association down-regulated and upregulated and the transcriptionally down-regulated lists.

Figure S3. Computational modelling to predict mRNAs that are insensitive to low levels of eEF2. Related to Figure 1F

The total rate of elongation is dependent on the processes of tRNA competition (that is completed by acceptance of the cognate tRNA and subsequent peptidyl transfer), and eEF2-catalysed ribosomal translocation. Under normal conditions, tRNA competition and translocation take a similar length of time for fast codons (which are decoded by abundant tRNAs). However, tRNA competition is much slower than translocation for slow codons (which are decoded by rarer tRNAs), as can be shown by detailed stochastic models of these processes.

(A) Mathematical modelling of *S. cerevisiae* peptidyl transfer times plotted for 3 individual codons, one of which is decoded by an abundant tRNA (AAA) whereas the other two are decoded by rare tRNAs (CGG and CCU). This constitutes the first step in translation elongation.

(B) The time for translocation, the second stage of elongation, is plotted as a function of eEF2 availability. Intracellular eEF2 concentrations are as for *S. cerevisiae*.

(C) To compare the effect of altered eEF2 availability on the total decoding time for an abundant and 2 rare codons we modified our model to simulate conditions of eEF2 ablation, where the translocation step becomes slower. These conditions disproportionally affect fast codons: at an eEF2 availability of 10% of full levels, fast codons are predicted to change their overall elongation rate by an order of magnitude, whereas slow codons change their overall elongation rate by less than two-fold. The change is expressed relative to the time taken at 100% eEF2 availability.

Figure S4. RTN3 is a slow elongating mRNA. **Related to Figure 2**

(A) HEK293 cells were treated with harringtonine to block initiation phase of translation. After 3 minutes, ribosome run-off from the mRNAs was blocked by addition of cycloheximide. Sucrose density gradient ultra-centrifugations were performed (as described in the materials and methods section). Plots show the distribution of RNA within subpolysomes (40S, 60S and 80S) and polysomes as indicated. RNA was isolated from fractions, which were then subjected to qPCR for β-Actin and RTN3 mRNAs. qPCR for 18S rRNA is used as a control. Error bars show standard deviation within a representative experiment.

(B) The efficiency of the ribosomes to run off the β-Actin and RTN3 mRNAs is calculated from 2 independent experiments. Error bars show standard deviation. The elongation rate of RTN3 mRNA is slower than the control in support of the model.

Figure S5. Protein changes upon cooling. Related to Figures 2,4 and 5

(A) HEK293 cells were treated with 100mg/ml cycloheximide, to block protein synthesis. After 2, 6 or 24 hours, protein extracts were analysed by Western Blots to assess the levels of RTN3 and c-Myc. RTN3 protein levels are unaffected by degradation at least up to 24 hours and therefore has a relatively long half life. In contrast c-Myc protein, which has a short half-life is rapidly degraded.

(B) Whole cell lysates from either HEK293, or SHSY5Y cells incubated at 37°C or 32°C for 24 hours, were analysed by western blot for poly-ubiquitinated proteins. Vinculin is used as a loading control. The data show that cooling reduces the level of steady state poly-ubiquitinated proteins. Bar graphs show quantification of poly-ubiquitin using data from 3 independent experiments. Error bars represent standard error.

(C) LV-shRNA-RTN3 accelerated synapse loss in the CA1 hippocampal region. Representative electron micrographs are shown, pseudo-coloured for ease of synapse identification: presynaptic, dark pink; postsynaptic light pink. Bar chart quantification is shown. Data represents are mean ± SEM. T-test *** $P < 0.0001$. Scale bar 1 μm

(D) In prion-infected mice total PrP and PrP^{Sc} levels do not alter after early cooling to 16–18°C following treatment with LV-shRNA-RTN3. PrP and PrP^{Sc} tested in terminal mice. Representative western blots are shown for 3 mice per temperature and time point and a single uninfected control mouse.

Top Networks

ID	Associated Network Functions	Score
1	Cell Death, Cancer, Endocrine System Disorders	49
2	Gastrointestinal Disease, Hepatic System Disease, Cell -To-Cell Signaling and Interaction	38
3	Hereditary Disorder, Metabolic Disease, Embryonic Development	31
4	Cell Cycle, Cell Death, Cell -To-Cell Signaling and Interaction	24
5	Cancer, Hereditary Disorder, Reproductive System Disease	20

Molecular and Cellular Functions

Name	p-value	# Molecules
Cellular Assembly and Organization	5.69E-05 - 2.96E-02	23
DNA Replication, Recombination, and Repair	5.69E-05 - 2.96E-02	10
Amino Acid Metabolism	1.10E-04 - 3.48E-02	5
Small Molecule Biochemistry	1.10E-04 - 3.48E-02	19
Cell Death	3.51E-04 - 3.54E-02	15

Top Canonical Pathways

Name	p-value	Ratio
G-Protein Coupled Receptor Signaling	4.98E-05	12/526 (0.023)
Relaxin Signaling	1.21E-03	5/157 (0.032)
Melanocyte Development and Pigmentation Signaling	1.68E-03	4/92 (0.043)
cAMP-mediated signaling	1.75E-03	6/219 (0.027)
mTOR Signaling	5.54E-03	5/209 (0.024)

Table S1. Related to Figure 1D and E and Figure S1.

Top significant networks, molecular functions and canonical pathways associated with the differentially expressed genes identified by Ingenuity Pathway Analysis.

Gene Name	logFC	P.Value
hsa-miR-346	0.41596256	0.04347048
hsa-miR-147	0.38747801	0.04702239
hsa-miR-424_MM1	0.261651	0.02224146
hsa-miR-551a	0.23395519	0.01667835
hsa-miR-590-5p	0.20676563	0.0436902
hsa-miR-130a_MM2	0.1867928	0.01700183
hsa-miR-223	0.14894424	0.03641048
hsa-miR-29c*	0.14838916	0.02312406
hsa-miR-607	0.14222777	0.04783652
hsa-miR-211	0.1219513	0.03575188
hsa-miR-371-3p_MM2	-0.14289755	0.02901324
hsa-miR-449b	-0.1587873	0.02097055
hsa-miR-320	-0.17020202	0.03939156
hsa-miR-20a	-0.1789382	0.02536781
hsa-miR-200b	-0.18473276	0.03840512
hsa-miR-518b	-0.23544463	0.04298777
hsa-miR-20b	-0.23634331	0.02701268
hsa-miR-296-5p	-0.2397331	0.00506562
hsa-miR-519e*	-0.24763121	0.02028128
hsa-miR-184	-0.44333136	0.04380508

Table S2. miRNA profiling. Related to Figure 1 and Figure S1

miRNA profiling was performed on samples isolated from cells incubated at either 32°C or 37 °C. No miRNA expression had more than a 2-fold difference between cooled and control.

Decreased polysomal association

Top Networks

ID	Associated Network Functions	Score
1	Cell Death, Liver Damage, Lipid Metabolism	49
2	Nucleic Acid Metabolism, Small Molecule Biochemistry, Cellular Assembly and Organization	32
3	Connective Tissue Disorders, Dermatological Diseases and Conditions, Developmental Disorder	32
4	Reproductive System Development and Function, Tissue Morphology, Tissue Development	21
5	Infectious Disease, Molecular Transport, Renal and Urological System Development and Function	8

Molecular and Cellular Functions

Name	p-value	# Molecules
DNA Replication, Recombination, and Repair	3.18E-04 - 4.64E-02	6
Carbohydrate Metabolism	2.88E-03 - 3.50E-02	7
Cell Cycle	3.95E-03 - 4.64E-02	6
Cell Death	3.95E-03 - 4.92E-02	18
Cell-To-Cell Signaling and Interaction	3.95E-03 - 4.26E-02	6

Top Canonical Pathways

Name	p-value	Ratio
Induction of Apoptosis by HIV1	1.71E-03	3/66 (0.045)
Mitochondrial Dysfunction	1.56E-02	3/174 (0.017)
CD27 Signaling in Lymphocytes	1.73E-02	2/57 (0.035)
Oxidative Phosphorylation	1.89E-02	3/159 (0.019)
Ubiquinone Biosynthesis	3.12E-02	2/114 (0.018)

Table S3. Translational profiling. Related to Figure 1 and Figure S1

Functional classification of genes down-regulated at translational level following cold shock. Top significant networks, molecular functions and canonical pathways associated with the differentially expressed genes identified by Ingenuity Pathway Analysis.

"Slow" mRNAs, less polysomally associated	Accession	Cellular Component (GO terms)	Biological Process (GO terms)
**PLXND1**	NM_015103	GO:0005887 = integral component of plasma membrane	GO:0071526 = semaphorin-plexin signaling pathway
**ATP5F1**	NM_001688	GO:0000276 = mitochondrial proton-transporting ATP synthase complex<br>GO:0005743 = mitochondrial inner membrane	GO:0015986 = ATP synthesis coupled proton transport
**RECK**	NM_021111	GO:0005886 = plasma membrane<br>GO:0031225 = anchored component of membrane	GO:0030198 = extracellular matrix organization
**RTN3**	NM_001265589	GO:0000139 = Golgi membrane<br>GO:0005789 = ER membrane	GO:0006915 = apoptotic process<br>GO:0071786 = ER tubular network organization
**FOXN3**	NM_001085471	GO:0005634 = nucleus	GO:0006355 = regulation of transcription
**NDUFA9**	NM_005002	GO:0005743 = mitochondrial inner membrane	GO:0006120 = mitochondrial electron transport
**JTB**	NM_006694	GO:0005887 = integral component of plasma membrane	GO:0008637 = apoptotic mitochondrial changes
**CHPT1**	NM_020244	GO:0000139 = Golgi membrane	GO:0006656 = phosphatidylcholine biosynthetic process
**NOG**	NM_005450	GO:0005576 = extracellular region	GO:0001701 = in utero embryonic development
**CYP2E1**	NM_000773	GO:0000139 = Golgi membrane<br>GO:0031227 = intrinsic component of ER membrane	GO:0019373 = epoxygenase P450 pathway
**HSPA5/BIP**	NM_005347	GO:0005788 = ER lumen<br>GO:0034663 = ER chaperone complex<br>GO:0030176 = integral component of ER membrane	GO:0030968 = ER unfolded protein response
**KDM1A**	NM_001009999	GO:0005634 = nucleus<br>GO:0005667 = transcription factor complex	GO:0000122 = negative regulation of transcription
**GPX4**	NM_001039847	GO:0005739 = mitochondrion<br>GO:0005829 = cytosol	GO:0006749 = glutathione metabolic process
**CPB1**	NM_001871	GO:0005576 = extracellular region	GO:0006508 = proteolysis
**SLC25A5**	NM_001152	GO:0005743 = mitochondrial inner membrane<br>GO:0005887 = integral component of plasma membrane	GO:1901029 = negative regulation of mitochondrial outer membrane permeabilization involved in apoptotic signaling pathway
**HAVCR1**	NM_001099414	GO:0016021 = integral component of membrane	GO:0046718 = viral entry into host cell
**SIVA1**	NM_006427	GO:0005634 = nucleus<br>GO:0005739 = mitochondrion	GO:1901030 = positive regulation of mitochondrial outer membrane permeabilization involved in apoptotic signaling pathway
**RNF13**	NM_007282	GO:0000139 = Golgi membrane<br>GO:0005789 = ER membrane<br>GO:0016021 = integral component of membrane	GO:0051865 = protein autoubiquitination
**CCT6A**	NM_001762	GO:0005832 = chaperonin-containing T-complex	GO:0006457 = protein folding
**AATK**	NM_004920	GO:0005783 = ER<br>GO:0044295 = axonal growth cone<br>GO:0016021 = integral component of membrane	GO:0007420 = brain development<br>GO:0030517 = negative regulation of axon extension

Table S4. Related to Figure 1 and Figure S1 and 2

A list of mRNAs less polysomally associated following cooling and that contained poorly optimised codons. Interestingly, many of these proteins are membrane associated.

	Relative to Ala-IGC	
	Average	St Dev
Ala-IGC	1.00	0.0000
Ala-hGC	3.58	0.2675
Arg-ICG	5.10	1.3591
Arg-yCG	3.32	0.7445
Arg-CCT	2.19	0.6331
Arg-TCT	2.76	0.3822
Asn-GTT	6.45	1.7786
Asp-GTC	10.61	4.4094
Cys-GCA	11.42	3.9288
Gln-yTG	10.19	3.4363
Glu-yTC	12.41	5.1321
Glu-TTC	8.71	2.4103
Gly-sCC	8.32	1.5825
Gly-TCC	11.21	3.4035
His-GTG	3.96	0.4647
Ile-rAT	4.50	0.2619
Ile-TAT	1.92	0.0034
Leu-wAG	4.61	0.7049
Leu-CAG	7.42	0.5317
Leu-CAA	4.77	0.4465
Leu-TAA1	1.46	0.0800
Lys-CTT	8.36	3.0412
Lys-TTT1	7.69	2.1075
Met-i	10.76	2.8043
Met-e	7.48	0.7467
Phe-GAA	7.73	0.0323
Pro-hGG	5.03	1.4005
Sec-TCA2	0.76	0.1401
Ser-CGA	3.46	1.1038
Ser-wGA	3.38	0.4407
Ser-GCT	4.88	1.7870
Thr-mGT	7.28	1.3873
Thr-TGT1	4.09	0.7747
Thr-CGT2	1.04	0.2128
Trp-CCA	6.83	0.3551
Tyr-GTA	3.35	1.0032
Val-mAC	9.12	1.2182
Val-TAC	3.46	0.2984

Table S5. tRNA concentration. Related to Figure 1 and Figure S3

A table to show the relative tRNA expression in HEK-293 cells.

Gene Name	Average Speed on initial 20 codon stretch (codons/sec)
'PLXND1'	1.362297336
'ATP5F1'	1.442855541
'RECK'	1.510570878
'RPAP3'	1.521085566
'RTN3'	1.565637314
'FOXN3'	1.586957801
'NDUFA9'	1.601096139
'JTB'	1.615165029
'CHPT1'	1.615872734
'NOG'	1.625053918
'CYP2E1'	1.649410682
'HSPA5'	1.652127552
'KDM1A'	1.661677058
'GPX4'	1.676822335
'CPB1'	1.688679086
'SLC25A5'	1.707429543
'KCNJ13'	1.708290697
'HAVCR1'	1.7099814

Table S6. Decoding speed of first 20 amino acids. Related to Figure 1, Figure 2 and Figure S4

Calculation of the decoding speed of the first 20 codons of the mRNAs identified as associated with a decreased number of ribosomes following cooling. The table lists the top slowest 25%. This window of 20 codons was chosen because ribosome speed on the first codons following the start codon can be analysed without taking ribosome queuing effects into account, which would reduce the confidence of any predictions on speed properties. This analysis would identify a minimal sub-set of mRNAs where expression levels are controlled via the elongation stage, although it would also miss many such mRNAs where the speed-limiting sequence is located further downstream.

Supplemental Experimental Procedures

Antibodies

Antibodies were purchased from Sigma (β-Actin), Cell Signalling (Noggin, GBBR1, LDHA, eIF2α, eEF2), Abcam (RBM3 (human), RTN3), Santa Cruz (GAPDH, c-Myc), Proteintech (CIRP, RBM3 (mouse)) and D-GEN (ICSM35 for PrP).

Plasmids

The 5'UTR of *RTN3* was amplified by PCR from HEK293 cell cDNA using the following primer pairs:

RTN3 5'UTR fw	gactagGCATGCGCGCTCGCGC
RTN3 5'UTR rv	catgccatgGGCTACGCGAGCGAG

These sequences were cloned into pGL3 (Promega) upstream of luciferase firefly between *Spe*I and *Nco*I restriction enzyme sites and were verified by sequencing.

Transfection

SiRNAs (siGENOME Non-Targeting siRNA Pool #1 and SMARTpool: ON-TARGETplus RBM3 siRNA, Dharmacon) transfection were performed using DharmaFECT 1 (Dharmacon) transfection reagent at a final concentration of 100 nM according to manufacturer instructions, after 24h cells were split and transfected with luciferase constructs 24h later.

DNA transfections: These were performed using Lipofectamine 2000 (Invitrogen) following the manufacturer's instructions and cells harvested 24 hours after transfection. Luciferase activity was measured using a dual luciferase assay system from Promega. Co-transfection with *Renilla* luciferase was used as an internal normalisation control.

Lentivirus: Mouse RBM3 isoform-2 (NM_001166410.1) overexpression was induced using the pLenti CAMKII (RBM3) Rsv (GFPBsd) plasmid [S1]. Mouse RTN3

(NM_053076.3) overexpression was induced with pLenti CAMKII (RBM3) Rsv (GFPBsd) plasmid (Gen Target Inc). The pLenti CAMKII (empty) Rsv (GFPBsd) plasmid was used as control.

RTN3 down regulation was achieved by using pLenti H1 shRNA (m RTN3) sequence #3 Rsv (GFPBsd). This plasmid contains the following shRNA-RTN3 sense, anti-sense and loop sequences (sequence #3:

5'TATGTTGGGATTGCCCGGGATcgagATCCCGGGCAATCCCAACATA 3'. The pLenty H1 shRNA (negative control) Rsv (GFPBsd) containing the sequence

5'-GTCTCCACGCGCAGTACATTTcgagAAATGTACTGCGCGTGGAGAC- 3'

was used as control. Viruses were used with a final titre of 0.6-1.5 x 108 TU. Prion infected mice were injected with lentiviruses at 2 w.p.i.

RT-PCR Primers

Mouse:

mRBM3 RT fw	ACTCTTCGTAGGAGGGCTCA
mRBM3 RT rev	CTCAGAGATAGGCCCAAAGC
mRTN3 RT fw	AGGCCTACTTGGATGTGGAC
mRTN3 RT rev	CAGCTTCAAGGAGTCAACCA

Human:

hACTIN RT fw	GTACCACTGGCATCGTGATGG
hACTIN RT rev	CCGCTCATTGCCAATGGTGAT
hGAPDH RT fw	GCACAGTCAAGGCCGAGAAT
hGAPDH RT rev	GCCTTCTCCATGGTGGTGAA
RBM3 h RT fw	GCTATGGGAGTGGCAGGTAT
RBM3 h RT rev	GTAGCGGTCATAACCACCCT

Protein synthesis rate determination

Cells were methionine starved for 30 minutes before addition of medium containing 40 µCi/ml Trans^{35}S -Label. After 30 minutes incubation, cells were harvested, washed and lysed in passive lysis buffer (Promega). Hippocampal slices were prepared. Slices were allowed to recover in normal artificial cerebrospinal fluid buffer while being

oxygenated either at 37°C or at 26°C for 1h, incubated with ^{35}S methionine label for 1h, then homogenized. Cells and slices lysates were centrifuged and protein precipitated from the supernatant with 10% trichloroacetic (TCA). Following washes with 70% IMS and 100% acetone, ^{35}S-methionine incorporation was determined by scintillation counting. Counts were normalised to total protein in the cell lysates as determined by Bradford assay (Bio-Rad).

Western Blotting

Cells and hippocampi lysed in protein lysis buffer (50 mM Tris, 150 mM NaCl, 1% Triton X-100, 1% Na deoxycholate, 0.1% SDS and 125 mM sucrose) supplemented with Phos-STOP and protease inhibitors (Roche), followed by centrifugation and quantification. PrP^{Sc} was detected after Proteinase K digestion. Lysates were subject to electrophoresis on SDS-polyacrylamide gels and the proteins transferred to PDVF or Nitrocellulose membranes (Bio-Rad). Blots were probed with the relevant antibodies and HRP-conjugated secondary antibodies, detected by ECL Prime western blotting detection reagent (GE Healthcare). Quantification was performed using ImageJ software.

Northern Blot analysis

RNA was separated by electrophoresis through a 1% formaldehyde-containing agarose gel and then transferred to Zeta-probe membrane (Bio-Rad) overnight in 20XSSC buffer and fixed by UV crosslinking. DNA hybridization probes were prepared from 50 ng DNA template and radiolabeled with αP^{32} CTP by random priming using Klenow enzyme (New England Biolabs). After overnight hybridisation with the radioactive probe in Church-Gilberts Solution (140 mM Na_2PO_4, 70 mM NaH_2PO_4, 7% SDS) at 55˚C, the membrane was washed twice with 2X SSC, 0.1% SDS, twice with 0.5X SSC, 0.1% SDS and twice with 0.1X SSC, 0.1% SDS.

Sucrose density gradient analysis

Cells were treated with cycloheximide at 100 µg/ml for 3 minutes prior to harvesting, pellets lysed in gradient buffer (300 mM NaCl, 15 mM MgCl2, 15 mM Tris pH 7.5 containing 1 mg/ml heparin sulphate and 100 µg/ml cycloheximide) plus 1% Triton X-100. The hippocampal tissues were dissected in ice-cold gradient buffer, and then homogenized in gradient buffer containing RNase inhibitors and 1.2% TritonX-100. Post nuclear supernatants were layered on 10-60% (w/v) sucrose gradients of gradient buffer. Gradients were centrifuged at 38,000 rpm for 2 hours at 4°C in a SW40Ti rotor (Beckman Coulter) then separated through a live optical density (OD) 254 nm UV spectrometer (Isco). Gradients were fractionated with continuous monitoring at 254 nm. Fractions of equal volume were collected directly into guandine-HCl, to give a final concentration of 5.8 M, and the RNA precipitated by addition of 1 volume of 100% ethanol. The RNA was resuspended in water and further purified by sodium acetate/ethanol precipitation.

Transcriptional Profiling

The human cDNA microarrays contained a set of total human cDNA clones. Total RNA from cells cultured either 24 hr at 37°C or at 32°C was extracted using TRIZOL (Invitrogen). Fluorescently labelled DNA probes were generated from equal proportions of RNAs (approximately 5µg) of 37°C RNA (Cy5) and 32°C RNA (Cy3) and hybridised to the arrays as described previously/ Microarray slides were scanned using a GenePix 4200B microarray scanner and GenePix Pro 6.0 software (Axon Instruments).

miRNA microarray

To investigate change in miRNA expression, miRNA microarray was carried out using a miRNA probe set from Exiqon (Exiqon, Vedbaek, Denmark) printed in-house.

RNA samples were labelled using FlashTagTM RNA labelling kit from Genisphere according to manufacturer instruction. Briefly 1.5ug of RNA was polyA tailed 15 min at 37°C followed by the labelling with a fluorophore (control with alexa 550 and coldshock with alexa-647) using T4 DNA ligase at room temperature for 30 min and then hybridised to the array at 52°C overnight. Following hybridisation slides were washed for 2 min in each wash solution (wash 1: 2X SSC, 0.2 %SDS; wash 2: 2X SSC; wash 3: 0.2X SSC), dried by centrifugation and scanned using a GenePix 4200B microarray scanner and GenePix Pro 6.0 software (Axon Instruments).

Analysis of Microarray Data

GenePix Pro 6.0 was used to quantify fluorescence intensities for individual spots on the microarray. All statistical analysis was performed in the statistical environment R, version 2.6.1 and the Limma package [S2]. Within-array normalisation using the print tip loess method and between-array normalisation using the scale method were performed on the data. Differentially expressed genes were then identified using the limFit and topTable functions. Genes which showed a fold change >2 and P-value < 0.05 were considered as potential candidates for altered distribution following coldshock at a statistically significant level. Once candidate genes were identified the sequence of the accession attached to each clone ID was search against the NCBI nucleotide database using nucleotide BLAST, with an E-value cut off of 0.001. The best match Refseq sequence(s) were chosen automatically for each clone ID. If no RefSeq hits were retrieved, non-RefSeq hits were checked manually for potential matches.

Immunoprecipitation reactions

Post-nuclear extracts were incubated with either anti-RBM3 antibody or IgG coated protein G magnetic beads which were washed (250 mM Tris pH 7.4, 300 mM NaCl, 5

mM $MgCl_2$, 0.5% NP40, 1 mM DTT and then resuspended in 200 µl buffer (50 mM Tris pH 7.4, 150 mM NaCl, 1 mM $MgCl_2$, 0.05% NP40) containing 0.1% SDS and 60 µg proteinase K. RNA was extracted using Trizol LS (Invitrogen) according to manufacturer instructions.

Supplemental References

S1. Peretti, D., Bastide, A., Radford, H., Verity, N., Molloy, C., Martin, M.G., Moreno, J.A., Steinert, J.R., Smith, T., Dinsdale, D., et al. (2015). RBM3 mediates structural plasticity and protective effects of cooling in neurodegeneration. Nature 518, 236-239.

S2. Smyth, G.K. (2004). Linear models and empirical bayes methods for assessing differential expression in microarray experiments. Stat Appl Genet Mol Biol *3*, Article3.
