# Supplementary material for: RTN3 Is a Novel Cold-Induced Protein and Mediates Neuroprotective Effects of RBM3
Source: Curr Biol. 2017 Mar 6;27(5):638–50. doi: 10.1016/j.cub.2017.01.047 (PMC5344685; doi:10.1016/j.cub.2017.01.047)
Supplement: Document S2. Article plus Supplemental Information [file mmc2.pdf]

Article

Current Biology

RTN3 Is a Novel Cold-Induced Protein and Mediates Neuroprotective Effects of RBM3

Graphical Abstract

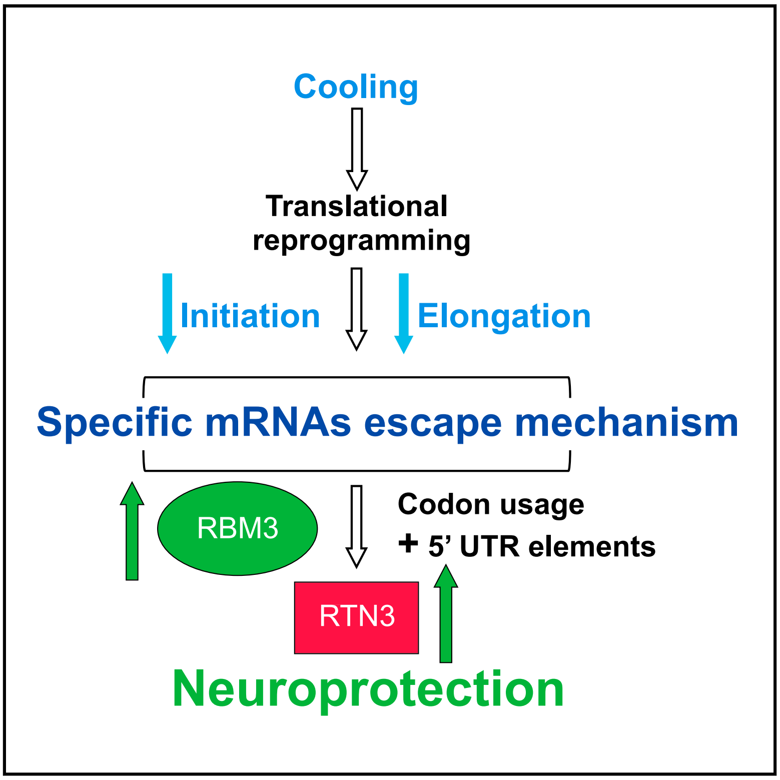

Authors

Amandine Bastide, Diego Peretti, John R.P. Knight, ..., C. Mark Smales, Giovanna R. Mallucci, Anne E. Willis

Correspondence

c.m.smales@kent.ac.uk (C.M.S.),
gm522@cam.ac.uk (G.R.M.),
aew5@le.ac.uk (A.E.W.)

In Brief

Therapeutic hypothermia is neuroprotective, and the cold shock protein RMB3 plays a critical role in mediating synaptic repair processes that accompany cooling. Bastide and Peretti et al. show that cooling selectively reprograms the translatome and identify RTN3 as a cold-induced protein that acts downstream of RBM3 in the neuroprotection pathway.

Highlights

- Cooling-induced reprogramming of the translatome increases synthesis of *RTN3*
- The neuroprotective protein RBM3 binds *RTN3* mRNA and drives its expression
- RTN3 overexpression prevents synaptic loss in mice with prion disease
- RTN3 expression is a mediator of RBM3-induced neuroprotection

Accession Numbers

E-MTAB-5437

Bastide et al., 2017, Current Biology *27*, 638–650
March 6, 2017 © 2017 The Author(s). Published by Elsevier Ltd.
http://dx.doi.org/10.1016/j.cub.2017.01.047

Current Biology
Article

RTN3 Is a Novel Cold-Induced Protein and Mediates Neuroprotective Effects of RBM3

Amandine Bastide,[1,4] Diego Peretti,[1,2,4] John R.P. Knight,[1] Stefano Grosso,[1] Ruth V. Spriggs,[1] Xavier Pichon,[1] Thomas Sbarrato,[1] Anne Roobol,[3] Jo Roobol,[3] Davide Vito,[3] Martin Bushell,[1] Tobias von der Haar,[3] C. Mark Smales,[3,*] Giovanna R. Mallucci,[1,2,*] and Anne E. Willis[1,5,*]
[1]Medical Research Council Toxicology Unit, Lancaster Road, Leicester LE1 9HN, UK
[2]Department of Clinical Neurosciences, University of Cambridge, Cambridge Biomedical Campus, Cambridge CB2 0AH, UK
[3]Centre for Molecular Processing and School of Biosciences, University of Kent, Canterbury, Kent CT2 7NJ, UK
[4]Co-first author
[5]Lead Contact
*Correspondence: c.m.smales@kent.ac.uk (C.M.S.), gm522@cam.ac.uk (G.R.M.), aew5@le.ac.uk (A.E.W.)
http://dx.doi.org/10.1016/j.cub.2017.01.047

SUMMARY

Cooling and hypothermia are profoundly neuroprotective, mediated, at least in part, by the cold shock protein, RBM3. However, the neuroprotective effector proteins induced by RBM3 and the mechanisms by which mRNAs encoding cold shock proteins escape cooling-induced translational repression are unknown. Here, we show that cooling induces reprogramming of the translatome, including the upregulation of a new cold shock protein, RTN3, a reticulon protein implicated in synapse formation. We report that this has two mechanistic components. Thus, *RTN3* both evades cooling-induced translational elongation repression and is also bound by RBM3, which drives the increased expression of RTN3. In mice, knockdown of RTN3 expression eliminated cooling-induced neuroprotection. However, lentivirally mediated RTN3 overexpression prevented synaptic loss and cognitive deficits in a mouse model of neurodegeneration, downstream and independently of RBM3. We conclude that RTN3 expression is a mediator of RBM3-induced neuroprotection, controlled by novel mechanisms of escape from translational inhibition on cooling.

INTRODUCTION

Therapeutic hypothermia is a powerful neuroprotectant, acting through multiple mechanisms, although the underlying pathways are not fully understood [1, 2]. We recently showed that the cold shock RNA-binding protein, RBM3, plays a critical role in mediating synaptic repair processes essential for neuroprotection in mouse models of neurodegenerative disease [3]. Thus, the inability to induce RBM3 expression in early disease results in defective structural synaptic plasticity and hence reduced regenerative capacity, leading to synapse loss and eventually neuronal loss. Inducing endogenous RBM3 expression in vivo through cooling, or by lentiviral-mediated overexpression, prevented synapse loss in prion and Alzheimer-type mice, rescued memory deficits, protected against neurodegeneration, and significantly prolonged survival [3]. How RBM3 mediates these effects is unknown, although it is likely to be related to its RNA chaperone function, as it facilitates selective mRNA translation following a number of cellular stresses, including cooling [4–6]. RBM3 is also implicated in protection against cell death [7, 8] and increases local protein synthesis at dendrites [9].

Upon cooling, the changes to the protein synthesis machinery are similar to those observed with other stress inducers (e.g., UVB exposure) [10, 11] and include phosphorylation of eukaryotic initiation factor 2 (eIF2) on the alpha subunit [12, 13]. However, cold shock differs because relieving eIF2α-mediated inhibition of translation is insufficient to restore protein synthesis rates [12]. Instead, the rapid reduction in protein synthesis that accompanies cooling [12–15] is a result of a decrease in translational elongation mediated by the phosphorylation of elongation factor 2 (eEF2) by elongation factor 2 kinase (eEF2K; a negative regulator of eEF2) [12]. Importantly, suppression of eEF2K and subsequent reactivation of eEF2 significantly increases the rate of protein synthesis rates in cooled cells [12], consistent with the concept that elongation is a major regulatory node under specific pathophysiological conditions [16, 17].

To examine the relationship between cold stress, RBM3 induction, and the modulation of mRNA translation for the synthesis of putative neuroprotective proteins, we investigated the post-transcriptional response to hypothermia in vitro and validated the data in vivo in a mouse model of neurodegeneration, in which we know cooling is protective, mediated by RBM3. We show that, following cold shock/cooling, the global decrease in protein synthesis rates is associated with selective reprogramming of the translatome. We find enhanced synthesis of specific proteins: not only the cold shock protein RBM3 (as predicted) but also of a number of proteins with a role in development and function of the nervous system, including reticulon 3 (RTN3). RTN3 has a role in synaptic plasticity and synapse formation [18, 19] and is thus a compelling candidate for the neuroprotective effects mediated by increased RBM3 expression. We find that both *RBM3* and *RTN3* evade translational repression and that

 © 2017 The Author(s). Published by Elsevier Ltd.
This is an open access article under the CC BY license (http://creativecommons.org/licenses/by/4.0/).

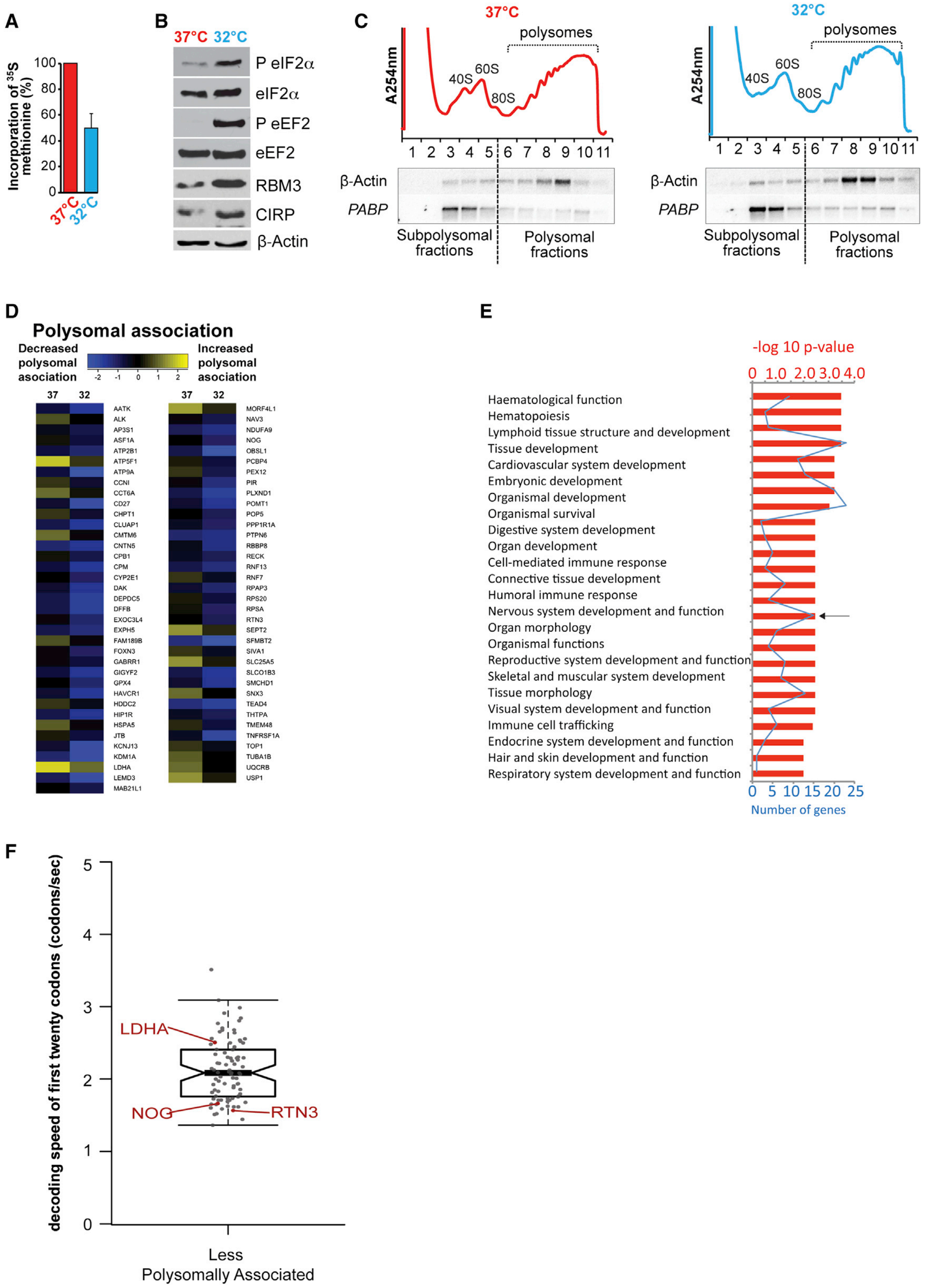

Figure 1. Mild Cooling Results in Translation Regulation of a Defined Set of Transcripts

(A) Protein synthesis rates determined by [^{35}S]-methionine label incorporation after 24 hr incubation of HEK293 cells at 32°C. Values were normalized to cells incubated at 37°C. Error bars represent SE within three independent experiments.

(B) HEK293 cells were incubated at 37°C or at 32°C for 24 hr and immunoblotted for RBM3 and CIRP, eIF2 alpha eEF2, and β-actin.

(legend continued on next page)

RBM3 binds *RTN3* mRNA and plays a major role in driving cooling-induced upregulation of RTN3 expression. Finally, we show that RTN3 expression, downstream of RBM3 induction, mediates cooling-induced neuroprotection in mice with neurodegenerative disease and importantly is neuroprotective even in the absence of cooling.

RESULTS

Cooling Induces Reprogramming of the Translatome

Both transcriptional and post-transcriptional control mechanisms are required for the overall response to cell stress [20]. In order to examine the genome-wide changes accompanying cooling, we incubated HEK293 cells at 32°C for 24 hr. This resulted in reduction in protein synthesis (Figure 1A) and phosphorylation of the translation initiation and elongation factors eIF2α and eEF2 (Figure 1B; in agreement with previous studies [12]). We chose HEK293 cells because the response to cooling is well documented [12, 15, 21] and, in addition, they express many markers associated with neuronal lineage [22]; thus, using this cell line increases the potential for the identification of cold-induced putative neuroprotective proteins. Transcriptional analyses of the cooled HEK293 cells showed that 119 genes were downregulated at the transcriptional level, with no significant increases in transcription of any mRNAs (Figure S1; Table S1). In addition, no differences greater than 2-fold were identified in the expression of microRNAs (miRNAs) (Table S2). These data support regulation of protein synthesis as an important mechanism for control of gene expression following cooling. We have shown previously that cooling reduces global rates of protein synthesis and importantly that elongation repression is the driver of this process (Figures 1A and 1B [12]). We hypothesize that, during cooling, specific mRNAs are able to evade a global reduction in translation elongation so that the expression of the corresponding proteins is maintained or even increased. However, identifying such mRNAs represents a technical challenge. Under conditions in which the initiation of translation is inhibited, the number of actively translating ribosomes decreases [10, 23] and polysome profiling can be used to identify those mRNAs that remain polysomally associated; this generally correlates with increased synthesis of the corresponding proteins [20]. However, under conditions in which elongation is inhibited, the number of polysomally associated ribosomes will stay the same or increase, and therefore it is difficult to identify mRNAs that either escape or are relatively insensitive to elongation slow down. Therefore, to identify proteins whose synthesis was increased during cooling, computational modeling was used in conjunction with polysome profiling.

Sucrose density gradient analysis was carried out on cooled samples to separate polysomes and the associated transcripts (Figure 1C), which were subsequently purified and analyzed by cDNA microarray. By microarray 71, mRNAs were identified that were associated with a decreased number of polysomes at 32°C (Figures 1D and S2; Tables S3 and S4). Importantly, ingenuity pathway analysis showed that there was significant enrichment for mRNAs that encode proteins that function in the nervous system and its development (15/71; marked by an arrow, Figure 1E). To predict which of these neuronal-related mRNAs were translated in cooled cells, we used a computational model of elongation control [24] generated by defining the intracellular concentration of ribosomes, translation factors, and tRNAs (Table S5). The model allows the speed of decoding to be estimated for any given open reading frame [25], assuming that decoding speed is not significantly limited by tRNA-independent parameters, such as mRNA secondary structure or modifications. We have previously shown that, despite this assumption, the model can be used to rank expression levels from multiple elongation-controlled mRNAs reliably [25]. The model predicts that, under eEF2 ablation, fast codons (which are decoded by abundant tRNAs) change their elongation rate by an order of magnitude, whereas slow codons are relatively unaffected (see Figures S3A–S3C).

We applied the model to the transcripts that encoded mRNAs with roles in neuronal processes identified by polysome profiling (Figure 1D) to analyze elongation over the initial 20 codons (Figure 1F; Table S6). Our analysis showed that a subset of mRNAs, including *RTN3* and *Noggin*, contained codons that require less abundant tRNAs in the 5′ end of the transcripts (Figure 1F), and our model predicted that these could escape the repression of elongation.

mRNAs Encoding a Subset of Neuronal Proteins Overcome Elongation Inhibition on Cooling

Western analysis showed that expression of Noggin and RTN3, and as expected RBM3, increased at 32°C, whereas GBBR1 and LDHA levels, which are encoded by mRNAs that contain codons requiring abundant tRNAs, were unchanged in both cell lines (Figure 2A). To confirm a post-transcriptional response, we examined mRNA expression changes of RTN3 and Noggin using qPCR (Figure 2B); there was a small reduction in the levels of Noggin, consistent with the transcriptional profiling data (Table S1; Figure S1) and no change for RTN3. There was an increase in the expression of RBM3 mRNA, in agreement with previous studies [4].

To confirm escape of translation elongation repression and identify a strong candidate protein with a neuroprotective function,

(C) Sucrose density gradient ultracentrifugations were performed from HEK293 cells incubated at 37°C or 32°C for 24 hr. Plots show the distribution of RNA within subpolysomes (40S, 60S, and 80S) and polysomes. Northern analysis was carried out on individual fractions, which were probed for β-actin or PABP.

(D) mRNAs from gradient fractions were pooled and subjected to cDNA microarray. The color scale represents the ratio of mRNA in subpolysome and polysome fractions, normalized $\log_2$ (polysome/subpolysome) value; yellow is polysome- and blue subpolysome-associated mRNAs.

(E) mRNAs that showed significant change in polysome/subpolysome (P/S) ratio on cooling were clustered into functional groups. Biological functions associated with decreased polysomal-associated transcripts, obtained from the ingenuity pathways analysis. Fisher's exact test was used to calculate a p value (threshold $p < 0.05$) for each biological function represented in the red bar chart. The blue line represents number of proteins per category.

(F) Predictive modeling of transcript-decoding speed was performed on the initial 20 codons of human transcript sequences from those that showed decreased polysomal association. The boxplot shows mRNAs that have a decrease in polysomal association and contain an initial 20 "slow" codons (e.g., RTN3 and Noggin [NOG]) compared to those that contain "fast" codons, such as LDHA.

See also Figures S1–S3 and Tables S1–S6.

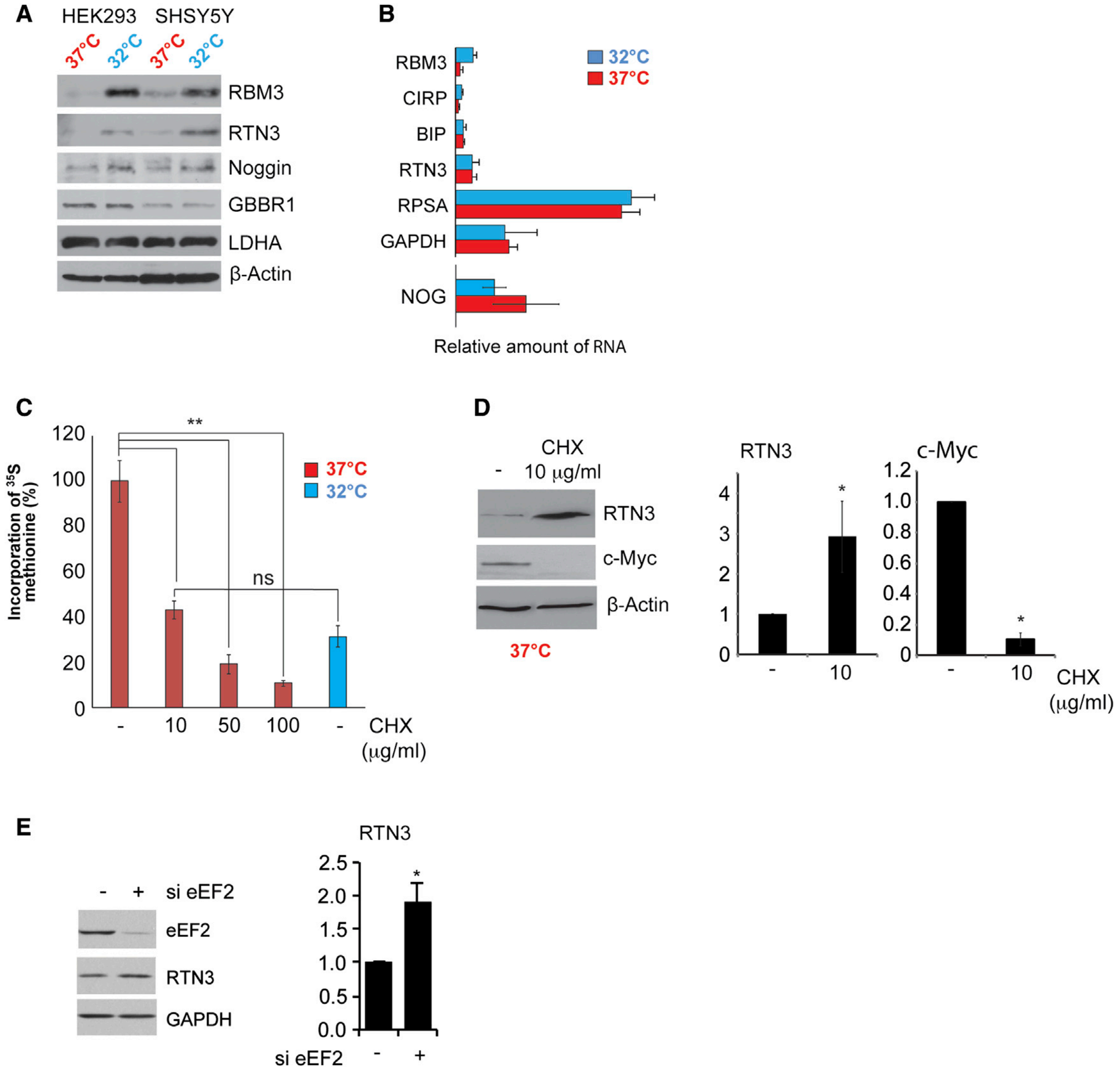

Figure 2. RTN3 Is Subject to Elongation Control

(A) Extracts from control or cooled HEK293 or SHSY5Y cells were immunoblotted for RTN3 and Noggin, GBBR1, and LDHA. β-actin is used as a loading control.

(B) qRT-PCR was performed on corresponding transcripts. Error bars represent 1 SD from the mean within three independent experiments. *GAPDH* was used as a control.

(C) Protein synthesis rates determined by [^{35}S]-methionine label incorporation after 24 hr incubation of HEK293 cells at 37°C with cycloheximide. Values were normalized to untreated cells. A two-tailed paired Student's t test was used to calculate statistical significance. Error bars represent SE within three independent experiments. **$p < 0.001$, all three conditions.

(D) Extracts from cells exposed to 10 μg/mL cycloheximide at 37°C were immunoblotted for RTN3, c-Myc, and β-actin. Error bars represent 1 SD from the mean within three independent experiments. *$p < 0.01$.

(E) eEF2 expression was reduced by siRNA, proteins extracted and immunoblotted with the antibodies shown. *GAPDH* was used as a loading control. Error bars represent 1 SD from the mean within three independent experiments. *$p < 0.01$.

See also Figures S4 and S5A.

we focused on RTN3, a protein that has known function in synaptogenesis in the adult nervous system [26–28] and a role in neuroprotection. To reduce elongation by an alternative method, we treated cells grown at 37°C with cycloheximide, which stalls the translocation step in the elongation cycle [29]. As expected, incubation with cycloheximide decreased global protein synthesis rates (Figure 2C) and reduced expression of c-Myc, which is known to have a short half-life of 20 min. However, there was increased RTN3 expression, consistent with our model's prediction that slowed elongation enhances synthesis of this protein (Figures 2D and S4).

To mimic the cooling-induced elongation block, we reduced eEF2 expression by RNAi (Figure 2E). This resulted in an increase in RTN3 levels, suggesting that the rate of elongation along *RTN3* mRNA is relatively unaffected by reduced availability of eEF2, in agreement with our model (Figure S3).

Whereas we have shown previously there is a small effect of cooling on the stability of specific proteins [21], RTN3 is

a relatively stable protein with a half-life of at least 24 hr, and therefore, any effects of turnover in the time frame of the experiment will be minimal (Figures S5A and S5B).

RBM3 Binds to RTN3 mRNA and Increases Its Translation through *trans*-Acting Effects on Initiation

Cooling also reduces the rate of translation initiation, via inhibitory phosphorylation of eIF2α (Figure 1A) [12], which compensates for the reduction in translation elongation, as fewer ribosomes will be available for initiation while they are limited by globally reduced elongation speeds. A similar phenomenon has been suggested to occur previously in yeast [25, 30]. Thus, we predict that, in order to increase their translation, transcripts also overcome the cooling-induced initiation block. This is likely to be driven by *trans*-acting factors acting upon *cis* elements within the transcripts. Because RBM3 is an RNA chaperone whose expression is increased in cooled cells [31, 32] and is known to mediate the neuroprotective effects of cooling [3], we hypothesized that cooling-induced RBM3 may act as a *trans*-acting factor promoting *RTN3* translation and that some of the neuroprotective effects of RBM3 may be mediated through RTN3.

To address whether RBM3 interacted with RTN3 mRNA, we carried out immunoprecipitation reactions, and data showed that RBM3 binds to *RTN3* mRNA in both HEK293 cells and in hippocampus of wild-type mice (Figures 3A and 3B). We then asked whether RBM3 expression affects RTN3 levels in HEK293 cells and mouse brain by transfecting with RBM3-expressing constructs or lentiviruses, respectively. In each case (Figures 3C and 3D), the data show that overexpression of RBM3 resulted in a dramatic increase in RTN3 protein expression, with no corresponding increase in *RTN3* transcript levels in vitro (Figure 3C) or in vivo (Figure 3D).

cis-Acting Elements in RTN3 Contribute to Evasion of the Initiation Block

To examine the role of *cis*-acting elements in 5′ UTR of *RTN3* in its post-transcriptional regulation by RBM3, we used a luciferase reporter construct containing the RTN3 5′ UTR (Figure 3E). At 25°C, there was a 5-fold increase in translation of messages containing the 5′ UTR of *RTN3*, compared to the control (Figure 3F). Further, overexpression of RBM3 resulted in a 5-fold induction of luciferase expression at 37°C (Figure 3Gi). In contrast, RNAi of RBM3 produced a small but significant reduction in luciferase activity (Figure 3Gii). Taken together, the data support a role for RBM3 in controlling RTN3 expression through the *RTN3* 5′ UTR cis-regulatory sequence.

Cooling Induces RTN3 Expression In Vivo through Post-transcriptional Mechanisms

Given the role of RBM3 in regulating RTN3 (Figures 2 and 3), we focused on RTN3 as a potential mediator of the neuroprotective effects of RBM3 induction. RTN3 is a strong candidate for this role. It is a member of the reticulon family of proteins, with multiple functions in the nervous system, including axon and neurite outgrowth [18, 19] and synapse formation [26–28]. It also has an indirect role in synaptic plasticity through its inhibition of BACE1, a secretase involved in cleavage of APP and a negative modulator of pCREB levels [28, 33, 34].

We tested whether RTN3 expression was increased in brain on cooling in vivo by inducing hypothermia in wild-type FVB mice using 5′ AMP, as described [3, 35]. We found the levels of both RBM3 and RTN3 increased on cooling (Figure 4Ai) without corresponding changes in respective mRNA levels (Figure 4Aii), consistent with post-transcriptional upregulation. Further, cooling induced a reduction in global protein synthesis rates to ~40% of control levels (Figure 4B), as observed in vitro (Figure 1A) (although the abundance of polysomes was not reduced to an equivalent extent [Figure 4C], again consistent with in vitro findings [Figure 1C] [12]). Knockdown of RBM3 in mice via lentivirally mediated RNAi significantly reduced the RTN3 increase on cooling (Figure 4D), confirming physiological relevance of this functional interaction and suggesting that RTN3 expression is downstream of RBM3 in cooling in vivo.

RTN3 Mediates Cooling-Induced Neuroprotective Effects of RBM3

We next asked to what extent RTN3 is neuroprotective in mice with neurodegenerative disease, using mice with prion disease, specifically tg37 mice [36] inoculated with Rocky Mountain Laboratory (RML) strain as in our previous studies [3, 37–41]. These mice overexpress prion protein (PrP) and have a rapid disease course, succumbing to disease in 12 weeks [36]. In these mice, synaptic loss is associated with the failure to induce RBM3 on cooling early in the disease course at 6 weeks post-inoculation (w.p.i.) [3], developing behavioral deficits at 9 w.p.i., with neuronal loss from 10 w.p.i. In general, terminal clinical signs appear at around 12 w.p.i.

We first confirmed that increased RTN3 expression is downstream of RBM3 in prion-diseased mice. Lentivirally mediated RNAi of *RTN3* reduced RTN3 levels in hippocampi (Figure 5A), but not RBM3 levels (Figure 5A). Further, RNAi of RTN3 did not affect high RBM3 levels induced by cooling while preventing, as predicted, the cold-induced rise in RTN3 (Figure 5B). This allowed us to address whether and to what extent RTN3 mediates the neuroprotective effects of RBM3. We found that lentivirally mediated RNAi of RTN3 in prion-infected mice abolished the protective effects of cooling on behavioral impairments (Figure 5C), accelerated neuronal loss (Figure 5D), and abolished the cooling-associated increase in survival in prion-infected mice (Figure 5E). Thus, reducing levels of RTN3 largely abolished the protective effects of high levels of cold-induced RBM3, supporting the conclusion that RTN3 is a major mediator of the effects of RBM3. Even in the absence of cooling, RNAi of RTN3 accelerated synapse loss in disease (Figures S5C and S5D), supporting a role for RTN3 in synapse maintenance and formation/plasticity. However, the exact mechanism remains unknown.

RTN3 Is Neuroprotective in Neurodegenerative Disease

RBM3 induction is profoundly neuroprotective in both prion and 5XFAD mice, effects that are abrogated if animals undergo knockdown of RBM3 [3] or of RTN3 (Figures 5C–5E). To address whether RTN3 is neuroprotective in the absence of cooling, we injected prion-infected tg37 mice with lentiviruses overexpressing RTN3 (LV-RTN3). This increased expression of RTN3 as expected (Figure 6A), importantly without increasing RBM3 expression, consistent with RTN3 being downstream of RBM3

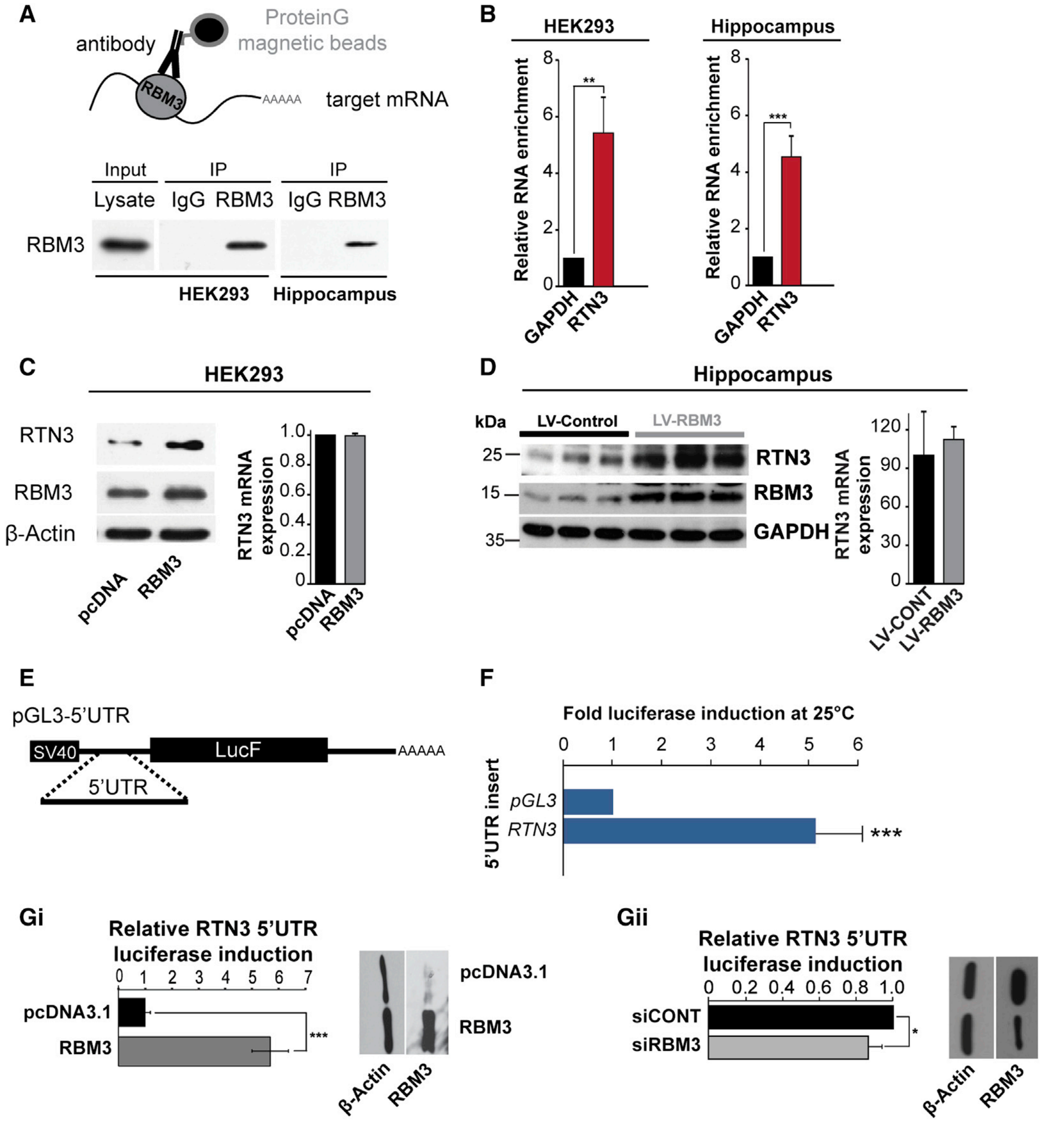

Figure 3. RTN3 Expression Is Downstream of RBM3

(A) Schematic representation of an RNA immunoprecipitation (RNA-IP) assay. Immunoblots of input lysate from HEK293 cells or hippocampus, immunoprecipitated with either rabbit IgG or RBM3 antibody, are shown.

(B) qRT-PCR was performed on RNA-IP samples using primers specific for human (HEK293) or mouse (hippocampus) samples. All values are normalized with respect to the initial RNA input material, and the enrichment is plotted relative to *GAPDH*. A two-tailed paired Student's t test was used to calculate statistical significance. Error bars represent 1 SD from the mean within three independent experiments. **$p < 0.01$; ***$p < 0.001$.

(C) HEK293 cells were transfected with an expression plasmid construct encoding RBM3 or a control plasmid, and extracts were immunoblotted for RTN3. β-actin was used as a loading control. RNA expression of RTN3 was assessed by qRT-PCR.

(D) Mouse hippocampi stereotaxically injected with lentivirus containing a construct to overexpress RBM3 and extracts were immunoblotted for RTN3 and GAPDH. qRT-PCR was used to assess the expression of RTN3.

(E) Schematic representation of the RTN3 containing plasmid constructs encoding firefly luciferase.

(F) HEK293 cells were transfected with construct containing the 5′ UTR of RTN3 and a Renilla luciferase control and incubated at either 37°C or 25°C for 24 hr. Firefly luciferase activity was calculated relative to Renilla luciferase for each condition and expressed as the fold induction from 37°C to 25°C.

(Gi) HEK293 cells were transfected with either control (pcDNA3.1) or RBM3 expression plasmid (pcDNA-RBM3) and then transfected with either RTN3 5′ UTR pGL3 or pGL3 and Renilla luciferase constructs and luciferase activity determined. A two-tailed paired Student's t test was used to calculate error. Error bars represent 1 SD from the mean within three independent experiments. ***$p < 0.001$.

(Gii) HEK293 cells were transfected with either control siRNA (siCONT) or *RBM3* siRNA (siRBM3) and then transfected with pGL3 and Renilla luciferase constructs. The fold repression from *RTN3* 5′ UTR pGL3 compared to the control pGL3 was calculated and normalized to siCONT transfection. A two-tailed paired Student's t test was used to calculate statistical significance. Error bars represent 1 SD from the mean within three independent experiments. *$p < 0.05$; **$p < 0.01$; ***$p < 0.001$.

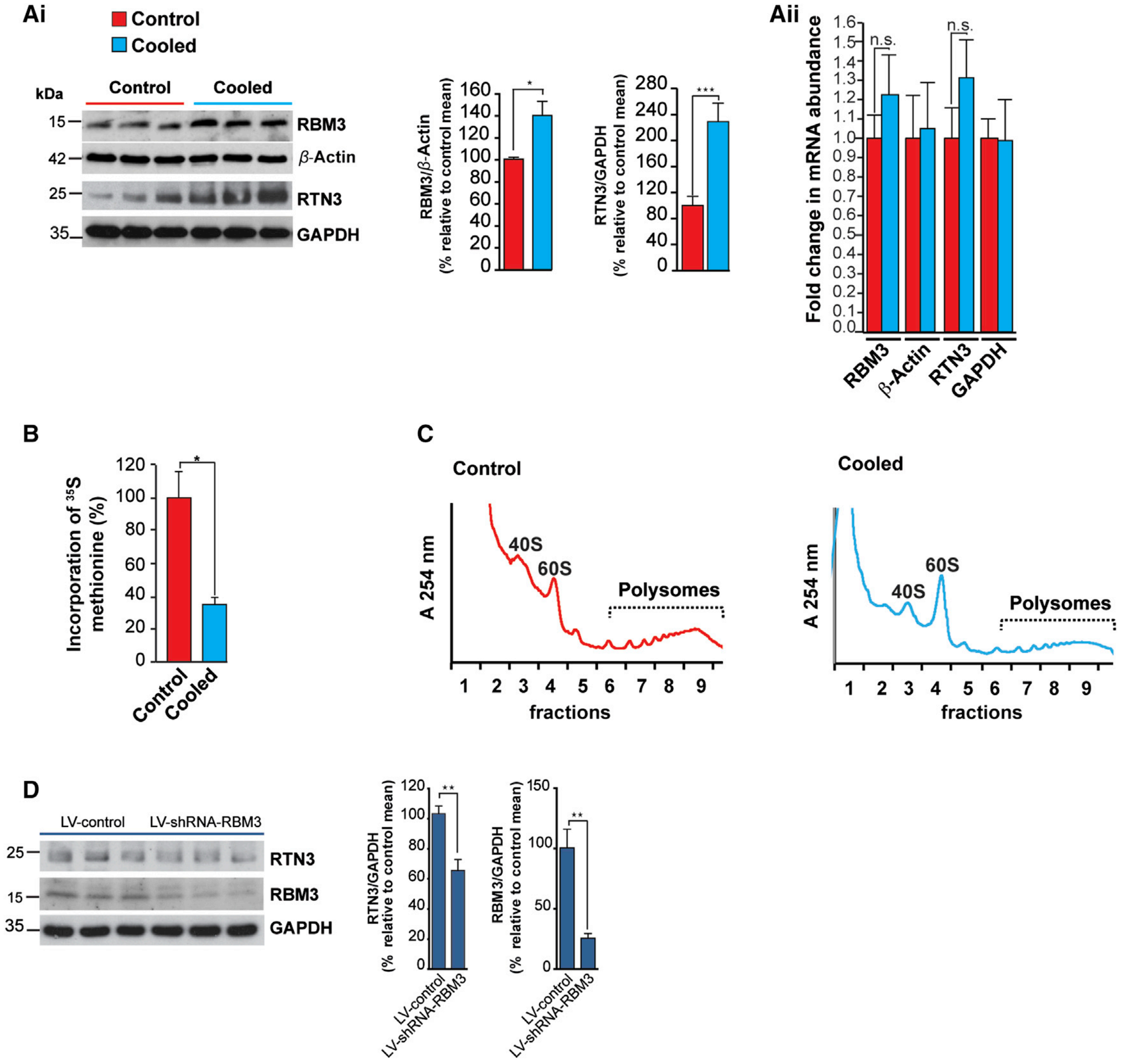

Figure 4. RBM3 Regulates RTN3 In Vivo

(Ai) Cooling increases RBM3 and RTN3 levels in hippocampi of wild-type mice. Representative western blots are shown. Bar graphs show quantification of RBM3 and RTN3 levels relative to β-actin and GAPDH, respectively. n = 5 control and 5 cooled mice for RBM3. n = 9 control mice and 9 cooled mice for RTN3. *$p < 0.05$; ***$p < 0.001$

(Aii) qRT-PCR of RNA isolated from hippocampi of cooled mice showed no significant change in the abundance of RBM3 or RTN3 mRNAs following cooling.

(B) Protein synthesis rates were determined by ^{35}S methionine incorporation into nascent protein using ex vivo hippocampus slices from cooled mice and control mice. A two-tailed paired Student's t test was used to calculate statistical significance. Error bars represent 1 SD from the mean within three independent experiments. *$p < 0.05$.

(C) Sucrose density gradient ultracentrifugation performed of cytoplasmic extracts from hippocampi from control and cooled mice. Absorbance plots show the distribution of RNA within subpolysomes (40S, 60S, and 80S) and polysomes.

(D) RTN3 induction on in vivo cooling is dependent on RBM3 protein expression. Knockdown of RBM3 resulted in a 38% decrease in RTN3 induction. n = 6 LV-control and 6 LV-shRNA-RBM3 mice. **$p < 0.01$.

(Figure 6A). To assess neuroprotective effects of RTN3 overexpression, we measured synapse number (Figure 6B), burrowing activity (Figure 6C), and neuron number in CA1 of hippocampus from diseased animals treated with LV-RTN3 compared to controls (Figure 6D) over the time course of disease. All of these parameters decline in the course of prion disease in the absence of intervention. Importantly, RTN3 overexpression restored synapse number to wild-type levels, markedly above levels seen in untreated mice at the same and later time points (Figure 6B), and prevented the decline in burrowing behavior (Figure 6C). RTN3 overexpression also conferred marked neuroprotection at the histological level, with very notable conservation of the CA1 pyramidal neuron layer (Figure 6D). Importantly, LV-RTN3 significantly increased survival of prion-infected mice (Figure 6E), recapitulating the effects of LV-RBM3 we previously described [3]. In our study of RBM3, we showed that onset of synaptic failure correlates with failure of induction of RBM3 at 6 w.p.i. [3]. And interestingly, RTN3 induction at this time point

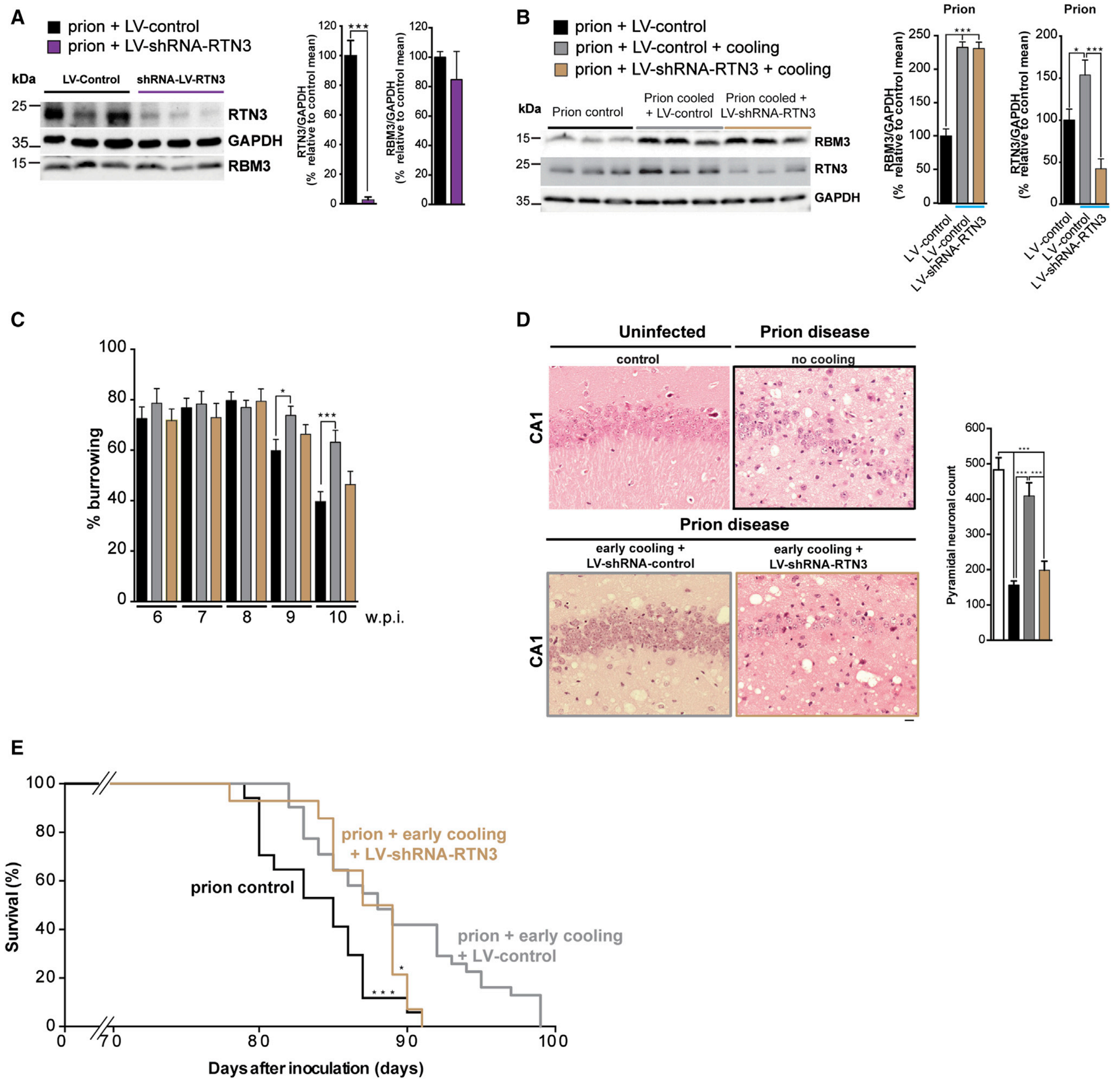

Figure 5. Lentiviral-Mediated Downregulation of Endogenous RTN3 Prevents Cooling-Induced RBM3-Mediated Neuroprotection

(A) LV-shRNA-RTN3 injected into hippocampi of prion-infected mice significantly reduces RTN3 protein levels compared to control shRNA (LV-shCONT). n = 6 prion+LV-control mice and 6 prion+LV-shRNA-RTN3 mice. Representative western blots and bar graphs quantification are shown.

(B) Western blot of RBM3 in LV-shRNA-RTN3-treated early-cooled prion mice shows no change in expression. n = 6 mice per experimental condition.

(C) The early-cooling-induced protection in burrowing behavior declines in LV-shRNA-RTN3 mice. Food pellet remaining in the tube measured after 2 hr is expressed in percentage burrowed. Graph bar with prion (black bars; n = 12 mice), prion + early cooling (gray bars; n = 20 mice), and prion + early cooling + LV-shRNA-RTN3 (light brown bars; n = 12 mice) is shown. One-way ANOVA with Tukey's post-test was used for multiple comparisons. *$p < 0.05$; ***$p < 0005$.

(D) Representative images of H&E-stained hippocampal sections from uninfected control (top left-hand panel), prion-infected mice (top right-hand panel), prion-infected mice treated with early cooling and LV-control (bottom left-hand panel), and prion-infected mice treated with early cooling and LV-shRNA-RTN3 (bottom right-hand panel). Prion-infected mice show extensive neuronal loss, with associated spongiosis, whereas early cooling treatment prevents neurodegeneration. This protection is abrogated with LV-shRNA-RTN3. The graph bar shows quantification of the average number of neurons for each condition in the CA1 area of hippocampus. n = 3 mice (white bar), 7 mice (black bar), 7 prion mice (bar), and 9 mice (light brown bar). One-way ANOVA and Brown-Forsythe test with Tukey's post hoc analysis for multiple comparisons were used. ***$p < 0.001$. The scale bar represents 50 μm.

(legend continued on next page)

is also lost (Figure 6F), supporting a functional relationship between these proteins in the context of disease and loss of synaptic structural plasticity.

Misfolded PrP levels were not affected by cooling or RTN3 expression, and levels were equivalent in all mice, precluding a mechanism of action via prion protein aggregation, consistent with our previous findings with RBM3 [3] (Figure S5D).

Thus, RTN3 overexpression results in neuroprotection at the level of synapse number, behavior, neuronal numbers, and increased survival, downstream and independently of cooling-mediated RBM3 induction.

DISCUSSION

The metabolic response to cooling is highly conserved [3, 35, 42]. The neuroprotective effects of hypothermia are essential for healthy brain function after hibernation and are widely exploited medically [43, 44]. However, relatively little is known about how global gene expression changes bring about these protective effects.

We have examined the genome-wide changes induced by cold stress by carrying out transcriptional, miRNA, and translational profiling on cells that were subjected to cooling. Our data show that specific induction of gene expression during cold stress is regulated at the level of translation with no significant transcriptional upregulation or changes in miRNA expression (Figures 1, S1, and S2; Tables S1 and S2).

Elongation rate control is the major determinant of global protein synthesis suppression upon cooling [12], but which transcripts are controlled in this way was unknown. We therefore generated a computational model to predict those messages that were particularly sensitive to regulation at this stage (Figure S3). According to our model, expression from transcripts that require abundant tRNAs would be dependent on eEF2 to maintain efficient elongation and protein expression. In contrast, mRNAs requiring rare tRNAs would be proportionally less affected by reductions in eEF2 availability and would be predicted to display either a small decrease or exhibit no net change in polysomal association upon cooling (Figure S3). In support of this hypothesis, the *RTN3* mRNA is decoded by rare tRNAs and, following cooling, exhibited reduced polysomal-associated yet increased protein expression (Figure 2). We showed that *cis*- and *trans*-acting factors were required for *RTN3* to overcome cooling-induced translation inhibition (Figure 3) and that the RNA chaperone RBM3 [6, 31, 32] was required (Figures 3 and 4).

We were interested in the functional consequence of RTN3 upregulation in response to cold shock. We have previously reported that RBM3 mediates the neuroprotective effects of cooling in mouse models of neurodegeneration and is necessary for maintenance of synaptic structural plasticity [3]. How it does this was not understood. We considered RTN3 to be a candidate neuroprotective protein specifically upregulated by RBM3 induction on cooling. In support of this, knockdown of RTN3 reduced synapse number at an earlier stage and abolished the neuroprotective effects of cooling in prion-diseased mice, despite cooling-induced increase in RBM3 levels (Figures 5 and S5C). Conversely, lentivirally mediated overexpression of RTN3 prevented synapse loss and neurodegeneration in prion-diseased mice (Figure 6), recapitulating the neuroprotective effects observed during RBM3 expression [3].

In conclusion, we propose that, following cooling, there is translational reprogramming, leading to the overexpression of specific cold-inducible proteins, including the known cold shock protein, RBM3, but also of RTN3. Critically, we show that induction of RTN3 is downstream of RBM3 expression, and our data suggest that RTN3 is a mediator of the RBM3-driven neuroprotective effects of cooling in prion-diseased mice, most likely through its multiple roles in the regulation of neurite outgrowth and regulation of synaptic plasticity. It is likely that RTN3 induction would mediate a similar neuroprotective role in other neurodegenerative conditions. Further, its inhibition of BACE1-mediated cleavage of APP could also contribute to neuroprotection in Alzheimer's pathology. We propose that the control of RTN3 expression through escape from inhibition of translation on cooling at the levels of initiation and elongation provides new targets for neuroprotective therapies in neurodegenerative disease.

EXPERIMENTAL PROCEDURES

All animal work was conducted according to UK Home Office Regulations. For details of antibodies, plasmids, and oligonucleotides, please see the Supplemental Experimental Procedures.

Cell Culture and Transfections

HEK293 cells were cultured under standard conditions in DMEM supplemented with 10% fetal bovine serum (FBS). For induction of cold stress, cells were incubated at 32°C or at 25°C for 24 hr before harvesting. Small interfering RNAs (siRNAs) were transfected using DharmaFECT 1 and plasmids with Lipofectamine 2000 (Invitrogen).

SDS-PAGE and Western Blotting

Western blots were performed as described previously [45] with modifications described in the Supplemental Experimental Procedures.

Determination of Protein Synthesis Rates

The rate of protein synthesis was measured by the incorporation of ^{35}S-methionine into trichloroacetic acid (TCA)-insoluble material as described previously [10]. Further details are provided in the Supplemental Experimental Procedures.

Sucrose Gradient Density Centrifugation and RNA Detection

Sucrose density gradient analysis was carried out as described [10]. Full details are provided in the Supplemental Experimental Procedures.

RNA Extraction

Total RNA extraction was performed using Trizol reagent (Invitrogen) according to the manufacturer's instructions.

Microarray Hybridization

The human cDNA microarrays were manufactured in Nottingham University. This custom cDNA microarray consists of 29,593 (32,448 total, including 2,855 control probes) oligonucleotide probes derived from MWG Human 30K slides A, B, and C. RNA from sucrose density gradient fractionation was

(E) Early cooling prolongs survival in prion-infected mice but is abolished by knockdown of RTN3. Kaplan-Meier plot; n = 25 cooled mice (gray line); n = 17 not cooled (black line); n = 14 cooled + shRNA of RTN3 (light brown line). One-way ANOVA with Tukey's post-test was used for multiple comparisons; not cooled versus cooled mice ***$p < 0.001$; not cooled versus cooled + shRNA of RTN3 n.s.; cooled mice versus cooled + shRNA of RTN3 *$p < 0.05$.
See also Figures 5C and 5D.

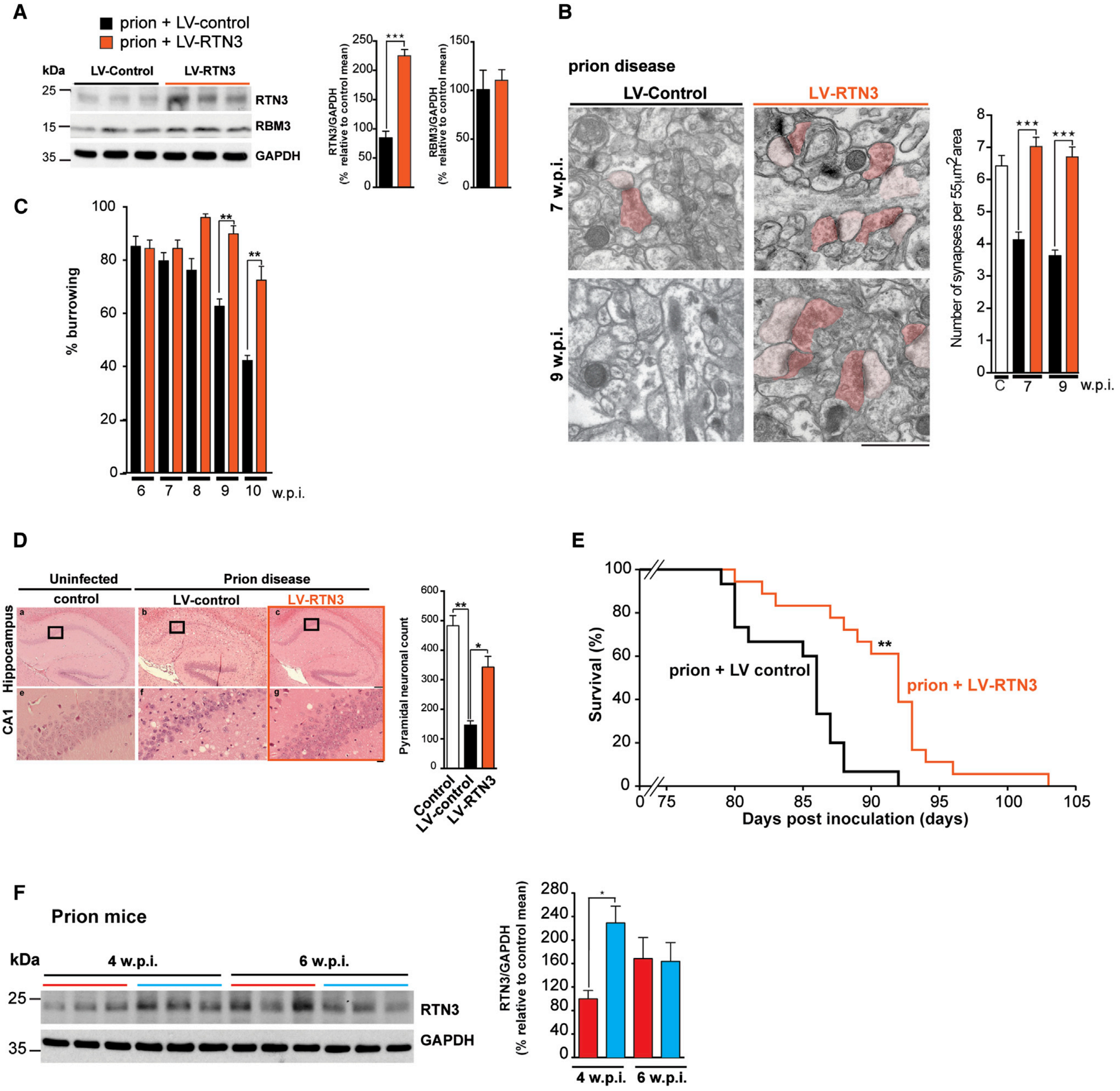

Figure 6. Enhanced RTN3 Expression Is Sufficient to Protect against Prion Disease in the Absence of Cooling

(A) LV-RTN3 delivery to hippocampi of prion-infected mice increases RTN3 in the absence of cooling compared to control lentiviral treatment (LV-control) and endogenous RBM3 (remain constant). n = 6 mice LV-control and 6 mice LV-shRNA-RTN3. Representative western blots and bar graphs quantification are shown.

(B) LV-RTN3 protected the deficit in synapse loss in prion-infected mice at 7 and 9 w.p.i. Representative electron micrographs are shown, pseudo-colored for ease of synapse identification: presynaptic, dark pink; postsynaptic, light pink. Bar chart quantification is shown. n = 93 images from three animals per condition. Data represent mean ± SEM; t test; ***$p < 0.0001$. The scale bar represents 1 µm.

(C) RTN3 overexpression prevented the decline in burrowing behavior of prion–infected mice. Food pellets remaining in the tube were measured after 2 hr and are expressed as percentage burrowed. Graph bar with prion (black bars; n = 14 mice) and prion + LV-RTN3 (orange bars; n = 20 mice) is shown. Kruskal-Wallis test with Dunn's multiple comparisons test; **$p < 0.01$.

(D) Representative images of H&E-stained hippocampal sections from uninfected control, prion-infected mice with LV-control, and prion-infected mice with LV-RTN3. The graph bar shows quantification of the average number of neurons for each condition in the CA1 area of hippocampus. n = 3 mice (white bar), 5 mice (black bar), and 15 mice (orange bar). One-way ANOVA and Brown-Forsythe test with Tukey's post hoc analysis for multiple comparisons were used. ***$p < 0.001$. The scale bars represent 400 µm (top row) and 50 µm (bottom row).

(legend continued on next page)

pooled into subpolysomal or polysomal fractions, labeled, and hybridized to the arrays as described previously [10]. Microarray slides were scanned using a GenePix 4200B microarray scanner and GenePix Pro 6.0 software (Axon Instruments).

Analysis of Microarray Data

GenePix Pro 6.0 was used to quantify fluorescence intensities for individual spots on the microarray. All statistical analysis was performed in the statistical environment R, version 2.6.1, and the Limma package [46].

Northern Blot

Northern analysis was performed as described previously [10]. Visualization and quantification of northern blot analysis was performed using a Molecular Imager FX phosphoimager and ImageJ software.

RNA-Protein Complex Immunoprecipitation

Post-nuclear extracts were incubated with either anti-RBM3 antibody or immunoglobulin G (IgG)-coated protein G magnetic beads and processed as described in the Supplemental Information.

Reverse Transcription and qPCR

Reverse transcription was carried out using random primers and Superscript III Reverse Transcriptase (Invitrogen) according to manufacturer instructions. qPCR was carried out using SensiFAST SYBR Lo-ROX Kit (Bioline) according to manufacturer instructions. Primers used are in the Supplemental Information.

Prion Infection of Mice

As described previously [37], hemizygous tg37 mice of both sexes were inoculated with 1% brain homogenate of Chandler/RML prions at 3–6 weeks of age. Control mice received 1% normal brain homogenate.

Induction of Hypothermia in Mice

FVB wild-type (WT) or hemizygous Tg37 mice weighing more than 20 g were cooled using 5′ AMP as described [3]. Prion-infected mice were injected with lentiviruses at 2 w.p.i. and subsequently cooled at 3 and 4 w.p.i.

Lentiviruses and Mice Stereotaxic Surgery

GenTarget generated lentiviral plasmids. Viruses were injected stereotaxically into the CA1 region of the hippocampus as described [3]; additional information is provided in the Supplemental Information.

Burrowing

Burrowing was performed as described [3]. Briefly, mice were placed in a cage with a tube full of pellets, which they “burrowed.” The extent of burrowing was assessed by the weight of pellets displaced in 2 hr.

Histology

Paraffin-embedded brains and pancreases were sectioned at 5 μm and stained with H&E as described [38, 39]. Neuronal counts were determined by quantifying NeuN-positive pyramidal CA1 neurons as described [39]. Synapses were counted in electron microscopy (EM) images of the stratum radiatum of the hippocampal CA1 region, blind. A synapse was defined as a structure with synaptic vesicles, synaptic cleft, and post-synaptic density, as described [3].

Computational Modeling

Translation elongation rates on human mRNAs were estimated using a published computational model [24]. The model was re-parameterized for the human decoding system using relative total tRNA abundances from [47] and a total tRNA concentration in HEK293 cells of 5.6 pg per cell, which was determined by comparing the staining intensity of the tRNA band in total RNA preps from HEK293 cells to the intensity of bands generated with known amounts of commercial yeast tRNA. Individual tRNA selection and translocation reactions were modeled in PRISM [48] using rate constants [49] and tRNA ratios for individual codons [24] as published.

ACCESSION NUMBERS

The accession number for the microarray data reported in this paper is ArrayExpress: E-MTAB-5437.

SUPPLEMENTAL INFORMATION

Supplemental Information includes Supplemental Experimental Procedures, five figures, and six tables and can be found with this article online at http://dx.doi.org/10.1016/j.cub.2017.01.047.

AUTHOR CONTRIBUTIONS

A.B., D.P., J.R.P.K., S.G., X.P., J.R., A.R., and D.V. conducted the experiments. R.V.S. and T.S. analyzed the data. T.v.d.H. generated the computational model and analyzed decoding speeds. A.E.W., M.B., G.R.M., and C.M.S. designed the experiments and wrote the paper.

ACKNOWLEDGMENTS

The work was funded by the BBSRC (BB/I019790/1; A.B., A.R., J.R., J.R.P.K., and R.V.S.), MRC (D.P., programme grant, 5TR50 to G.R.M.; S.G., programme funding, 5TR00 to A.E.W.), and Wellcome Trust (2010847/Z/16/Z; S.G., collaborative award to A.E.W., G.R.M., C.M.S., and T.v.d.H.).

Received: October 17, 2016
Revised: December 21, 2016
Accepted: January 23, 2017
Published: February 23, 2017

REFERENCES

1. Delhaye, C., Mahmoudi, M., and Waksman, R. (2012). Hypothermia therapy: neurological and cardiac benefits. J. Am. Coll. Cardiol. *59*, 197–210.
2. Yenari, M.A., and Han, H.S. (2012). Neuroprotective mechanisms of hypothermia in brain ischaemia. Nat. Rev. Neurosci. *13*, 267–278.
3. Peretti, D., Bastide, A., Radford, H., Verity, N., Molloy, C., Martin, M.G., Moreno, J.A., Steinert, J.R., Smith, T., Dinsdale, D., et al. (2015). RBM3 mediates structural plasticity and protective effects of cooling in neurodegeneration. Nature *518*, 236–239.
4. Wellmann, S., Bührer, C., Moderegger, E., Zelmer, A., Kirschner, R., Koehne, P., Fujita, J., and Seeger, K. (2004). Oxygen-regulated expression of the RNA-binding proteins RBM3 and CIRP by a HIF-1-independent mechanism. J. Cell Sci. *117*, 1785–1794.
5. Fedorov, V.B., Goropashnaya, A.V., Tøien, O., Stewart, N.C., Chang, C., Wang, H., Yan, J., Showe, L.C., Showe, M.K., and Barnes, B.M. (2011). Modulation of gene expression in heart and liver of hibernating black bears (Ursus americanus). BMC Genomics *12*, 171.
6. Liu, X., Wang, M., Chen, H., Guo, Y., Ma, F., Shi, F., Bi, Y., and Li, Y. (2013). Hypothermia protects the brain from transient global ischemia/reperfusion by attenuating endoplasmic reticulum response-induced apoptosis through CHOP. PLoS ONE *8*, e53431.

(E) LV-RTN3 significantly lengthened survival of prion-infected mice. Kaplan-Meier plot (orange; n = 18) compared to LV-control prion-infected mice (black; n = 15); t test; **p < 0.01.
(F) Induction of RTN3 fails at 6 w.p.i. in prion-diseased mice. Blue lines above the western blots are samples from cooled mice, whereas red lines denote control mice. Bar graphs show quantification of RTN3 levels relative to GAPDH at 4 and 6 w.p.i. prion disease, blue bars represent quantification from cooled mice, and red bars from control mice. n = 9 mice per condition. *p < 0.05. Data are mean ± SEM.

7. Chip, S., Zelmer, A., Ogunshola, O.O., Felderhoff-Mueser, U., Nitsch, C., Bührer, C., and Wellmann, S. (2011). The RNA-binding protein RBM3 is involved in hypothermia induced neuroprotection. Neurobiol. Dis. *43*, 388–396.

8. Tong, G., Endersfelder, S., Rosenthal, L.M., Wollersheim, S., Sauer, I.M., Bührer, C., Berger, F., and Schmitt, K.R. (2013). Effects of moderate and deep hypothermia on RNA-binding proteins RBM3 and CIRP expressions in murine hippocampal brain slices. Brain Res. *1504*, 74–84.

9. Smart, F., Aschrafi, A., Atkins, A., Owens, G.C., Pilotte, J., Cunningham, B.A., and Vanderklish, P.W. (2007). Two isoforms of the cold-inducible mRNA-binding protein RBM3 localize to dendrites and promote translation. J. Neurochem. *101*, 1367–1379.

10. Powley, I.R., Kondrashov, A., Young, L.A., Dobbyn, H.C., Hill, K., Cannell, I.G., Stoneley, M., Kong, Y.W., Cotes, J.A., Smith, G.C., et al. (2009). Translational reprogramming following UVB irradiation is mediated by DNA-PKcs and allows selective recruitment to the polysomes of mRNAs encoding DNA repair enzymes. Genes Dev. *23*, 1207–1220.

11. Somers, J., Wilson, L.A., Kilday, J.P., Horvilleur, E., Cannell, I.G., Pöyry, T.A., Cobbold, L.C., Kondrashov, A., Knight, J.R., Puget, S., et al. (2015). A common polymorphism in the 5′ UTR of ERCC5 creates an upstream ORF that confers resistance to platinum-based chemotherapy. Genes Dev. *29*, 1891–1896.

12. Knight, J.R., Bastide, A., Roobol, A., Roobol, J., Jackson, T.J., Utami, W., Barrett, D.A., Smales, C.M., and Willis, A.E. (2015). Eukaryotic elongation factor 2 kinase regulates the cold stress response by slowing translation elongation. Biochem. J. *465*, 227–238.

13. Roobol, A., Roobol, J., Bastide, A., Knight, J.R., Willis, A.E., and Smales, C.M. (2015). p58IPK is an inhibitor of the eIF2alpha kinase GCN2 and its localization and expression underpin protein synthesis and ER processing capacity. Biochem. J. *465*, 213–225.

14. Roobol, A., Carden, M.J., Newsam, R.J., and Smales, C.M. (2009). Biochemical insights into the mechanisms central to the response of mammalian cells to cold stress and subsequent rewarming. FEBS J. *276*, 286–302.

15. Roobol, A., Roobol, J., Carden, M.J., Bastide, A., Willis, A.E., Dunn, W.B., Goodacre, R., and Smales, C.M. (2011). ATR (ataxia telangiectasia mutated- and Rad3-related kinase) is activated by mild hypothermia in mammalian cells and subsequently activates p53. Biochem. J. *435*, 499–508.

16. Richter, J.D., and Coller, J. (2015). Pausing on polyribosomes: make way for elongation in translational control. Cell *163*, 292–300.

17. Faller, W.J., Jackson, T.J., Knight, J.R., Ridgway, R.A., Jamieson, T., Karim, S.A., Jones, C., Radulescu, S., Huels, D.J., Myant, K.B., et al. (2015). mTORC1-mediated translational elongation limits intestinal tumour initiation and growth. Nature *517*, 497–500.

18. Kumamaru, E., Kuo, C.H., Fujimoto, T., Kohama, K., Zeng, L.H., Taira, E., Tanaka, H., Toyoda, T., and Miki, N. (2004). Reticulon3 expression in rat optic and olfactory systems. Neurosci. Lett. *356*, 17–20.

19. Matsuzaki, F., Shirane, M., Matsumoto, M., and Nakayama, K.I. (2011). Protrudin serves as an adaptor molecule that connects KIF5 and its cargoes in vesicular transport during process formation. Mol. Biol. Cell *22*, 4602–4620.

20. Spriggs, K.A., Bushell, M., and Willis, A.E. (2010). Translational regulation of gene expression during conditions of cell stress. Mol. Cell *40*, 228–237.

21. Knight, J.R., Bastide, A., Peretti, D., Roobol, A., Roobol, J., Mallucci, G.R., Smales, C.M., and Willis, A.E. (2016). Cooling-induced SUMOylation of EXOSC10 down-regulates ribosome biogenesis. RNA *22*, 623–635.

22. Stepanenko, A.A., and Dmitrenko, V.V. (2015). HEK293 in cell biology and cancer research: phenotype, karyotype, tumorigenicity, and stress-induced genome-phenotype evolution. Gene *569*, 182–190.

23. Bushell, M., Stoneley, M., Kong, Y.W., Hamilton, T.L., Spriggs, K.A., Dobbyn, H.C., Qin, X., Sarnow, P., and Willis, A.E. (2006). Polypyrimidine tract binding protein regulates IRES-mediated gene expression during apoptosis. Mol. Cell *23*, 401–412.

24. Chu, D., and von der Haar, T. (2012). The architecture of eukaryotic translation. Nucleic Acids Res. *40*, 10098–10106.

25. Chu, D., Kazana, E., Bellanger, N., Singh, T., Tuite, M.F., and von der Haar, T. (2014). Translation elongation can control translation initiation on eukaryotic mRNAs. EMBO J. *33*, 21–34.

26. Chang, K., Seabold, G.K., Wang, C.Y., and Wenthold, R.J. (2010). Reticulon 3 is an interacting partner of the SALM family of adhesion molecules. J. Neurosci. Res. *88*, 266–274.

27. Laurén, J., Hu, F., Chin, J., Liao, J., Airaksinen, M.S., and Strittmatter, S.M. (2007). Characterization of myelin ligand complexes with neuronal Nogo-66 receptor family members. J. Biol. Chem. *282*, 5715–5725.

28. Deng, M., He, W., Tan, Y., Han, H., Hu, X., Xia, K., Zhang, Z., and Yan, R. (2013). Increased expression of reticulon 3 in neurons leads to reduced axonal transport of β site amyloid precursor protein-cleaving enzyme 1. J. Biol. Chem. *288*, 30236–30245.

29. Schneider-Poetsch, T., Ju, J., Eyler, D.E., Dang, Y., Bhat, S., Merrick, W.C., Green, R., Shen, B., and Liu, J.O. (2010). Inhibition of eukaryotic translation elongation by cycloheximide and lactimidomycin. Nat. Chem. Biol. *6*, 209–217.

30. Shah, P., Ding, Y., Niemczyk, M., Kudla, G., and Plotkin, J.B. (2013). Rate-limiting steps in yeast protein translation. Cell *153*, 1589–1601.

31. Sureban, S.M., Ramalingam, S., Natarajan, G., May, R., Subramaniam, D., Bishnupuri, K.S., Morrison, A.R., Dieckgraefe, B.K., Brackett, D.J., Postier, R.G., et al. (2008). Translation regulatory factor RBM3 is a proto-oncogene that prevents mitotic catastrophe. Oncogene *27*, 4544–4556.

32. Liu, Y., Hu, W., Murakawa, Y., Yin, J., Wang, G., Landthaler, M., and Yan, J. (2013). Cold-induced RNA-binding proteins regulate circadian gene expression by controlling alternative polyadenylation. Sci. Rep. *3*, 2054.

33. He, W., Lu, Y., Qahwash, I., Hu, X.Y., Chang, A., and Yan, R. (2004). Reticulon family members modulate BACE1 activity and amyloid-beta peptide generation. Nat. Med. *10*, 959–965.

34. Chen, Y., Huang, X., Zhang, Y.W., Rockenstein, E., Bu, G., Golde, T.E., Masliah, E., and Xu, H. (2012). Alzheimer's β-secretase (BACE1) regulates the cAMP/PKA/CREB pathway independently of β-amyloid. J. Neurosci. *32*, 11390–11395.

35. Zhang, J., Kaasik, K., Blackburn, M.R., and Lee, C.C. (2006). Constant darkness is a circadian metabolic signal in mammals. Nature *439*, 340–343.

36. Mallucci, G.R., Ratté, S., Asante, E.A., Linehan, J., Gowland, I., Jefferys, J.G.R., and Collinge, J. (2002). Post-natal knockout of prion protein alters hippocampal CA1 properties, but does not result in neurodegeneration. EMBO J. *21*, 202–210.

37. Mallucci, G., Dickinson, A., Linehan, J., Klöhn, P.C., Brandner, S., and Collinge, J. (2003). Depleting neuronal PrP in prion infection prevents disease and reverses spongiosis. Science *302*, 871–874.

38. Moreno, J.A., Halliday, M., Molloy, C., Radford, H., Verity, N., Axten, J.M., Ortori, C.A., Willis, A.E., Fischer, P.M., Barrett, D.A., and Mallucci, G.R. (2013). Oral treatment targeting the unfolded protein response prevents neurodegeneration and clinical disease in prion-infected mice. Sci. Transl. Med. *5*, 206ra138.

39. Moreno, J.A., Radford, H., Peretti, D., Steinert, J.R., Verity, N., Martin, M.G., Halliday, M., Morgan, J., Dinsdale, D., Ortori, C.A., et al. (2012). Sustained translational repression by eIF2α-P mediates prion neurodegeneration. Nature *485*, 507–511.

40. Mallucci, G.R., White, M.D., Farmer, M., Dickinson, A., Khatun, H., Powell, A.D., Brandner, S., Jefferys, J.G., and Collinge, J. (2007). Targeting cellular prion protein reverses early cognitive deficits and neurophysiological dysfunction in prion-infected mice. Neuron *53*, 325–335.

41. White, M.D., Farmer, M., Mirabile, I., Brandner, S., Collinge, J., and Mallucci, G.R. (2008). Single treatment with RNAi against prion protein rescues early neuronal dysfunction and prolongs survival in mice with prion disease. Proc. Natl. Acad. Sci. USA *105*, 10238–10243.

CellPress

42. Daniels, I.S., Zhang, J., O'Brien, W.G., 3rd, Tao, Z., Miki, T., Zhao, Z., Blackburn, M.R., and Lee, C.C. (2010). A role of erythrocytes in adenosine monophosphate initiation of hypometabolism in mammals. J. Biol. Chem. *285*, 20716–20723.
43. van der Worp, H.B., Sena, E.S., Donnan, G.A., Howells, D.W., and Macleod, M.R. (2007). Hypothermia in animal models of acute ischaemic stroke: a systematic review and meta-analysis. Brain *130*, 3063–3074.
44. Jena, A.B., Romley, J.A., Newton-Cheh, C., and Noseworthy, P. (2012). Therapeutic hypothermia for cardiac arrest: real-world utilization trends and hospital mortality. J. Hosp. Med. *7*, 684–689.
45. West, M.J., Stoneley, M., and Willis, A.E. (1998). Translational induction of the c-myc oncogene via activation of the FRAP/TOR signalling pathway. Oncogene *17*, 769–780.
46. Smyth, G.K. (2004). Linear models and empirical bayes methods for assessing differential expression in microarray experiments. Stat. Appl. Genet. Mol. Biol. *3*, Article3.
47. Dittmar, K.A., Goodenbour, J.M., and Pan, T. (2006). Tissue-specific differences in human transfer RNA expression. PLoS Genet. *2*, e221.
48. Kwiatkowska, M., Norman, G., and Parker, D. (2011). PRISM 4.0: verification of probabilistic real-time systems. In Computer Aided Verification, *Volume 6806*, G. Gopalakrishnan, and S. Qadeer, eds. (Springer), pp. 585–591.
49. Fluitt, A., Pienaar, E., and Viljoen, H. (2007). Ribosome kinetics and aa-tRNA competition determine rate and fidelity of peptide synthesis. Comput. Biol. Chem. *31*, 335–346.

Current Biology, Volume *27*

Supplemental Information

RTN3 Is a Novel Cold-Induced Protein and Mediates Neuroprotective Effects of RBM3

Amandine Bastide, Diego Peretti, John R.P. Knight, Stefano Grosso, Ruth V. Spriggs, Xavier Pichon, Thomas Sbarrato, Anne Roobol, Jo Roobol, Davide Vito, Martin Bushell, Tobias von der Haar, C. Mark Smales, Giovanna R. Mallucci, and Anne E. Willis

Figure S1

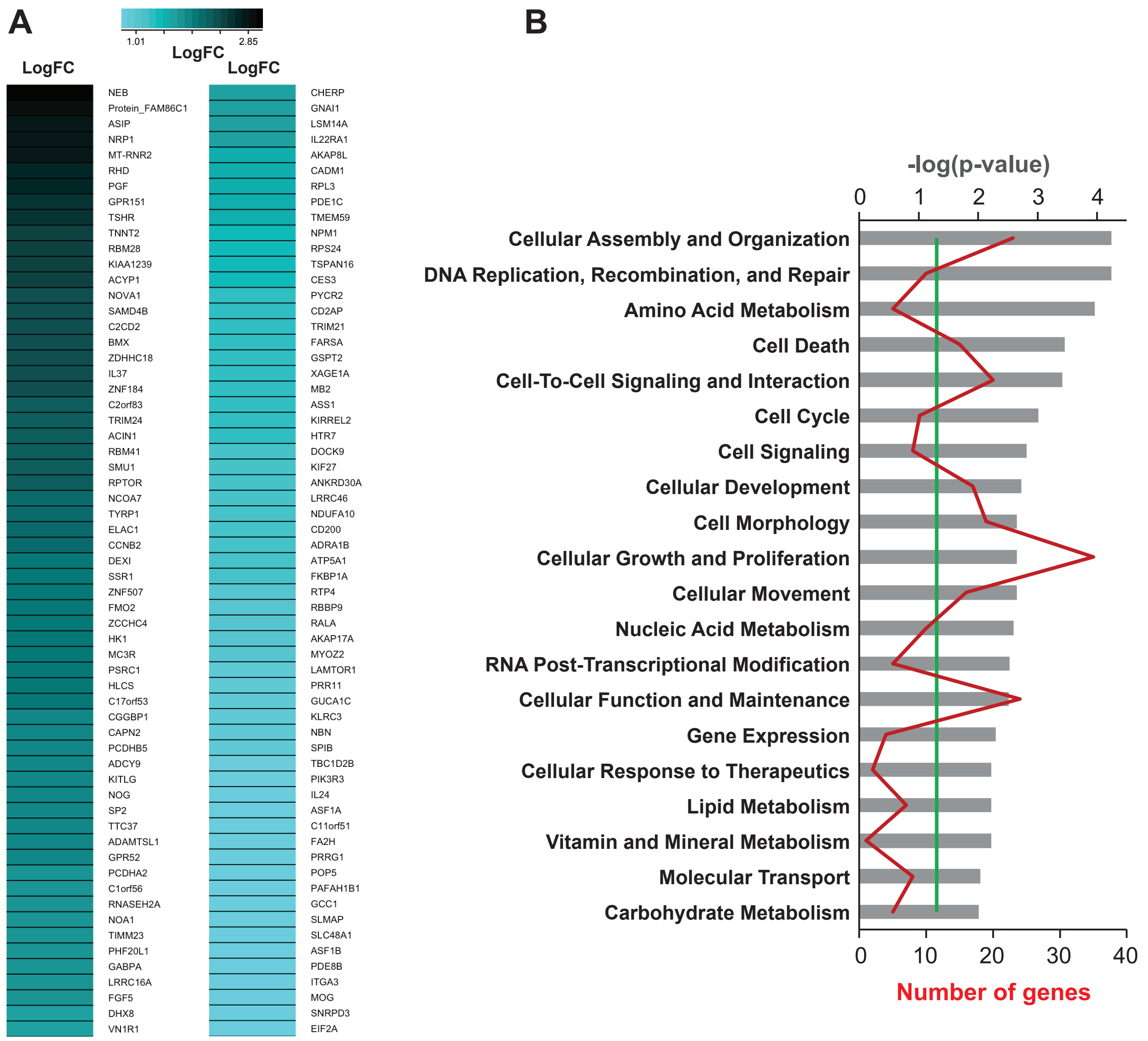
A
1.01
2.85
LogFC
LogFC
LogFC
NEB
Protein_FAM86C1
ASIP
NRP1
MT-RNR2
RHD
PGF
GPR151
TSHR
TNNT2
RBM28
KIAA1239
ACYP1
NOVA1
SAMD4B
C2CD2
BMX
ZDHHC18
IL37
ZNF184
C2orf83
TRIM24
ACIN1
RBM41
SMU1
RPTOR
NCOA7
TYRP1
ELAC1
CCNB2
DEXI
SSR1
ZNF507
FMO2
ZCCHC4
HK1
MC3R
PSRC1
HLCS
C17orf53
CGGBP1
CAPN2
PCDHB5
ADCY9
KITLG
NOG
SP2
TTC37
ADAMTSL1
GPR52
PCDHA2
C1orf56
RNASEH2A
NOA1
TIMM23
PHF20L1
GABPA
LRRC16A
FGF5
DHX8
VN1R1
CHERP
GNAI1
LSM14A
IL22RA1
AKAP8L
CADM1
RPL3
PDE1C
TMEM59
NPM1
RPS24
TSPAN16
CES3
PYCR2
CD2AP
TRIM21
FARSA
GSPT2
XAGE1A
MB2
ASS1
KIRREL2
HTR7
DOCK9
KIF27
ANKRD30A
LRRC46
NDUFA10
CD200
ADRA1B
ATP5A1
FKBP1A
RTP4
RBBP9
RALA
AKAP17A
MYOZ2
LAMTOR1
PRR11
GUCA1C
KLRC3
NBN
SPIB
TBC1D2B
PIK3R3
IL24
ASF1A
C11orf51
FA2H
PRRG1
POP5
PAFAH1B1
GCC1
SLMAP
SLC48A1
ASF1B
PDE8B
ITGA3
MOG
SNRPD3
EIF2A
B
-log(p-value)
0
1
2
3
4
Cellular Assembly and Organization
DNA Replication, Recombination, and Repair
Amino Acid Metabolism
Cell Death
Cell-To-Cell Signaling and Interaction
Cell Cycle
Cell Signaling
Cellular Development
Cell Morphology
Cellular Growth and Proliferation
Cellular Movement
Nucleic Acid Metabolism
RNA Post-Transcriptional Modification
Cellular Function and Maintenance
Gene Expression
Cellular Response to Therapeutics
Lipid Metabolism
Vitamin and Mineral Metabolism
Molecular Transport
Carbohydrate Metabolism
0
10
20
30
40
Number of genes

Figure S2

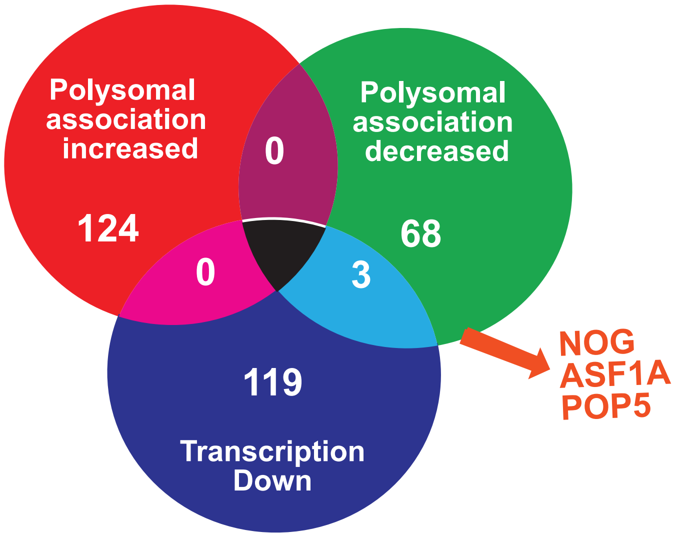
Polysomal association increased
124
0
Polysomal association decreased
68
0
3
119
Transcription Down
NOG
ASF1A
POP5

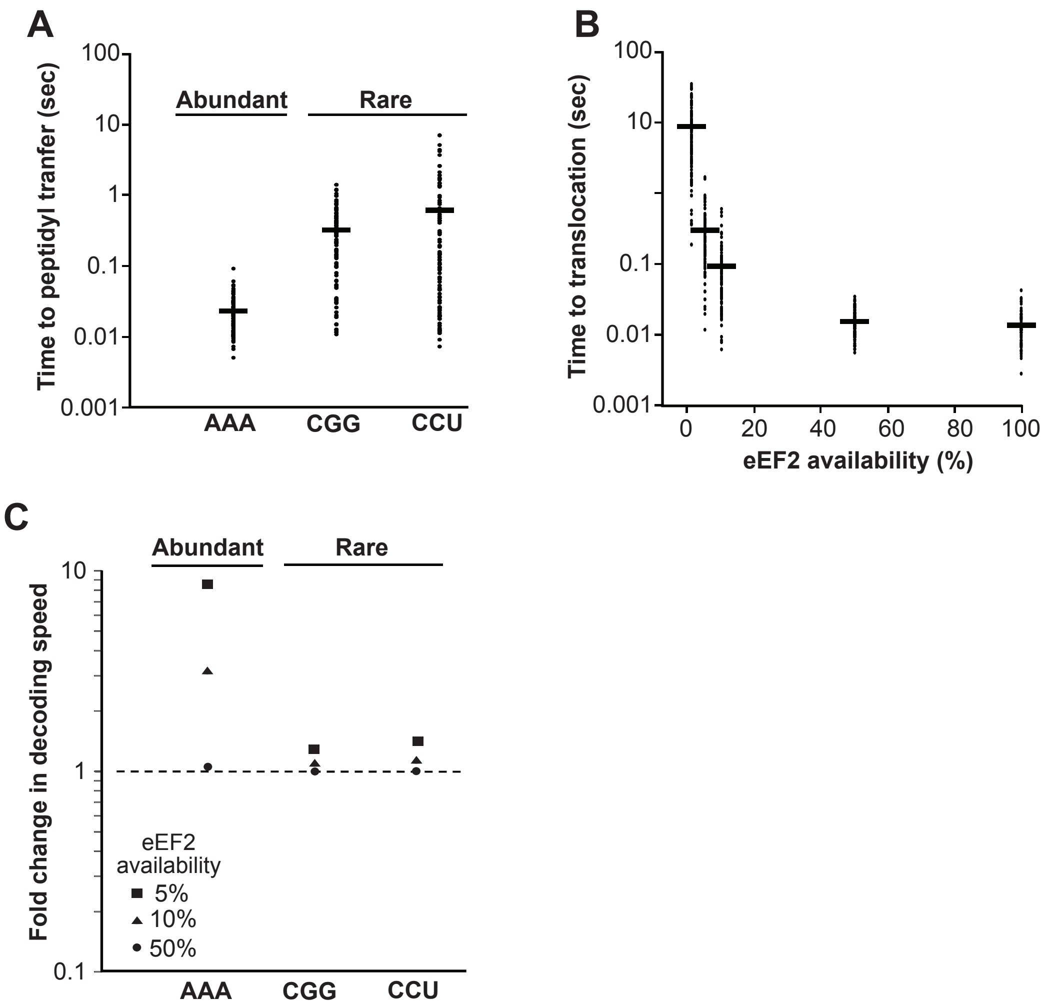
A
Time to peptidyl tranfer (sec)
100
10
1
0.1
0.01
0.001
Abundant
Rare
AAA
CGG
CCU
B
Time to translocation (sec)
100
10
0.1
0.01
0.001
0
20
40
60
80
100
eEF2 availability (%)
C
Fold change in decoding speed
Abundant
Rare
10
1
0.1
eEF2
availability
5%
10%
50%
AAA
CGG
CCU

Figure S4

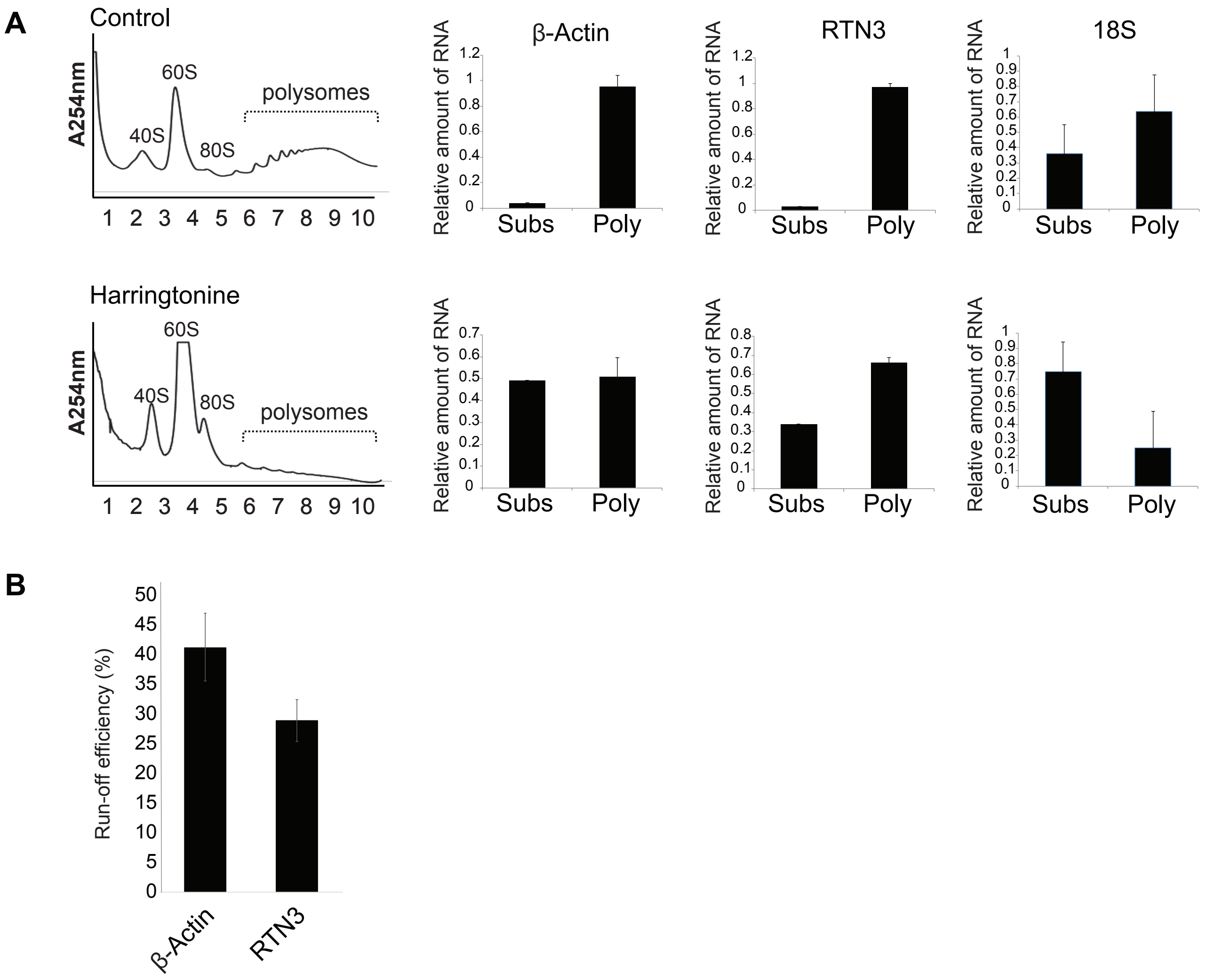
A
Control
A254nm
40S
60S
80S
polysomes
1 2 3 4 5 6 7 8 9 10
Harringtonine
β-Actin
RTN3
18S
Relative amount of RNA
Subs
Poly
B
Run-off efficiency (%)
β-Actin
RTN3

Figure S5

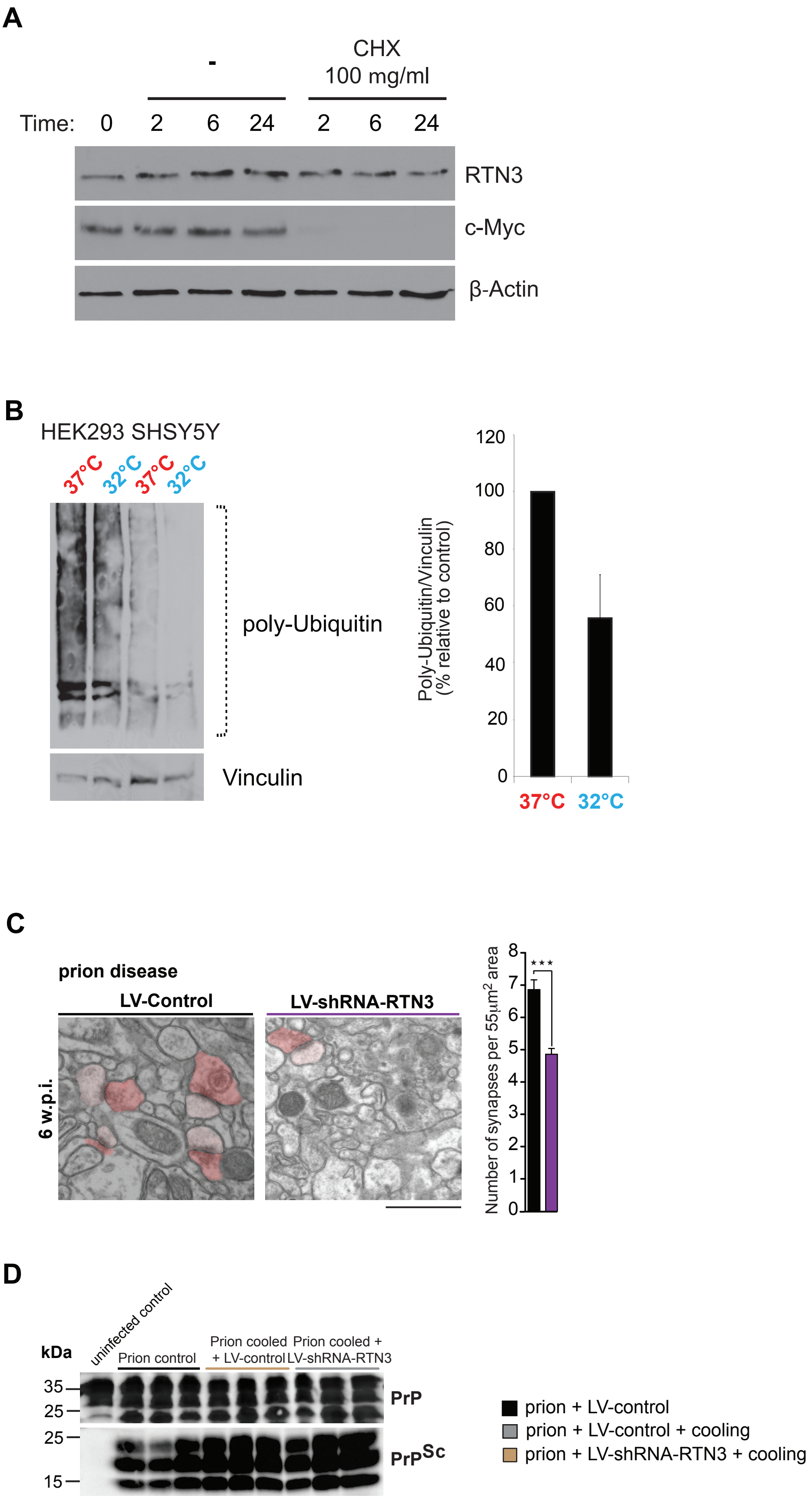
A
-
CHX
100 mg/ml
Time: 0 2 6 24 2 6 24
RTN3
c-Myc
β-Actin
B
HEK293 SHSY5Y
37°C 32°C 37°C 32°C
poly-Ubiquitin
Vinculin
Poly-Ubiquitin/Vinculin
(% relative to control)
0
20
40
60
80
100
120
37°C
32°C
C
prion disease
LV-Control
LV-shRNA-RTN3
6 w.p.i.
Number of synapses per 55µm² area
0
1
2
3
4
5
6
7
8
***
D
kDa
uninfected control
Prion control
Prion cooled
+ LV-control
Prion cooled +
LV-shRNA-RTN3
35
25
25
15
PrP
PrPSc
prion + LV-control
prion + LV-control + cooling
prion + LV-shRNA-RTN3 + cooling

Figure S1. Transcriptional profiling following mild coldshock. Related to Figure 1

(A) Genes shown are those with a 2-fold change and a statistically significant difference between control and cooled conditions. Values represent the mean, normalised $\log_2$ ratio of transcript level in cold-shock versus control conditions.

(B) Functional classification of genes down-regulated at transcriptional level following cold shock. Biological functions associated with regulated genes, obtained from the Ingenuity Pathways Analysis. p-value (threshold $p < 0.05$) for each biological function are represented by the grey bar chart, number of genes per category is represented by the purple line.

Figure S2. Venn diagram of to show genome-wide changes on cold shock. Related to Figure 1, Figure S1 and Tables S1-S3

The Venn diagram shows the number of overlapping genes between polysomal association down-regulated and upregulated and the transcriptionally down-regulated lists.

Figure S3. Computational modelling to predict mRNAs that are insensitive to low levels of eEF2. Related to Figure 1F

The total rate of elongation is dependent on the processes of tRNA competition (that is completed by acceptance of the cognate tRNA and subsequent peptidyl transfer), and eEF2-catalysed ribosomal translocation. Under normal conditions, tRNA competition and translocation take a similar length of time for fast codons (which are decoded by abundant tRNAs). However, tRNA competition is much slower than translocation for slow codons (which are decoded by rarer tRNAs), as can be shown by detailed stochastic models of these processes.

(A) Mathematical modelling of *S. cerevisiae* peptidyl transfer times plotted for 3 individual codons, one of which is decoded by an abundant tRNA (AAA) whereas the other two are decoded by rare tRNAs (CGG and CCU). This constitutes the first step in translation elongation.

(B) The time for translocation, the second stage of elongation, is plotted as a function of eEF2 availability. Intracellular eEF2 concentrations are as for *S. cerevisiae*.

(C) To compare the effect of altered eEF2 availability on the total decoding time for an abundant and 2 rare codons we modified our model to simulate conditions of eEF2 ablation, where the translocation step becomes slower. These conditions disproportionally affect fast codons: at an eEF2 availability of 10% of full levels, fast codons are predicted to change their overall elongation rate by an order of magnitude, whereas slow codons change their overall elongation rate by less than two-fold. The change is expressed relative to the time taken at 100% eEF2 availability.

Figure S4. RTN3 is a slow elongating mRNA. Related to Figure 2

(A) HEK293 cells were treated with harringtonine to block initiation phase of translation. After 3 minutes, ribosome run-off from the mRNAs was blocked by addition of cycloheximide. Sucrose density gradient ultra-centrifugations were performed (as described in the materials and methods section). Plots show the distribution of RNA within subpolysomes (40S, 60S and 80S) and polysomes as indicated. RNA was isolated from fractions, which were then subjected to qPCR for β-Actin and RTN3 mRNAs. qPCR for 18S rRNA is used as a control. Error bars show standard deviation within a representative experiment.

(B) The efficiency of the ribosomes to run off the β-Actin and RTN3 mRNAs is calculated from 2 independent experiments. Error bars show standard deviation. The elongation rate of RTN3 mRNA is slower than the control in support of the model.

Figure S5. Protein changes upon cooling. Related to Figures 2,4 and 5

(A) HEK293 cells were treated with 100mg/ml cycloheximide, to block protein synthesis. After 2, 6 or 24 hours, protein extracts were analysed by Western Blots to assess the levels of RTN3 and c-Myc. RTN3 protein levels are unaffected by degradation at least up to 24 hours and therefore has a relatively long half life. In contrast c-Myc protein, which has a short half-life is rapidly degraded.

(B) Whole cell lysates from either HEK293, or SHSY5Y cells incubated at 37°C or 32°C for 24 hours, were analysed by western blot for poly-ubiquitinated proteins. Vinculin is used as a loading control. The data show that cooling reduces the level of steady state poly-ubiquitinated proteins. Bar graphs show quantification of poly-ubiquitin using data from 3 independent experiments. Error bars represent standard error.

(C) LV-shRNA-RTN3 accelerated synapse loss in the CA1 hippocampal region. Representative electron micrographs are shown, pseudo-coloured for ease of synapse identification: presynaptic, dark pink; postsynaptic light pink. Bar chart quantification is shown. Data represents are mean ± SEM. T-test *** $P < 0.0001$. Scale bar 1 μm

(D) In prion-infected mice total PrP and PrP^{Sc} levels do not alter after early cooling to 16–18°C following treatment with LV-shRNA-RTN3. PrP and PrP^{Sc} tested in terminal mice. Representative western blots are shown for 3 mice per temperature and time point and a single uninfected control mouse.

Top Networks

ID	Associated Network Functions	Score
1	Cell Death, Cancer, Endocrine System Disorders	49
2	Gastrointestinal Disease, Hepatic System Disease, Cell -To-Cell Signaling and Interaction	38
3	Hereditary Disorder, Metabolic Disease, Embryonic Development	31
4	Cell Cycle, Cell Death, Cell -To-Cell Signaling and Interaction	24
5	Cancer, Hereditary Disorder, Reproductive System Disease	20

Molecular and Cellular Functions

Name	p-value	# Molecules
Cellular Assembly and Organization	5.69E-05 - 2.96E-02	23
DNA Replication, Recombination, and Repair	5.69E-05 - 2.96E-02	10
Amino Acid Metabolism	1.10E-04 - 3.48E-02	5
Small Molecule Biochemistry	1.10E-04 - 3.48E-02	19
Cell Death	3.51E-04 - 3.54E-02	15

Top Canonical Pathways

Name	p-value	Ratio
G-Protein Coupled Receptor Signaling	4.98E-05	12/526 (0.023)
Relaxin Signaling	1.21E-03	5/157 (0.032)
Melanocyte Development and Pigmentation Signaling	1.68E-03	4/92 (0.043)
cAMP-mediated signaling	1.75E-03	6/219 (0.027)
mTOR Signaling	5.54E-03	5/209 (0.024)

Table S1. Related to Figure 1D and E and Figure S1.

Top significant networks, molecular functions and canonical pathways associated with the differentially expressed genes identified by Ingenuity Pathway Analysis.

Gene Name	logFC	P.Value
hsa-miR-346	0.41596256	0.04347048
hsa-miR-147	0.38747801	0.04702239
hsa-miR-424_MM1	0.261651	0.02224146
hsa-miR-551a	0.23395519	0.01667835
hsa-miR-590-5p	0.20676563	0.0436902
hsa-miR-130a_MM2	0.1867928	0.01700183
hsa-miR-223	0.14894424	0.03641048
hsa-miR-29c*	0.14838916	0.02312406
hsa-miR-607	0.14222777	0.04783652
hsa-miR-211	0.1219513	0.03575188
hsa-miR-371-3p_MM2	-0.14289755	0.02901324
hsa-miR-449b	-0.1587873	0.02097055
hsa-miR-320	-0.17020202	0.03939156
hsa-miR-20a	-0.1789382	0.02536781
hsa-miR-200b	-0.18473276	0.03840512
hsa-miR-518b	-0.23544463	0.04298777
hsa-miR-20b	-0.23634331	0.02701268
hsa-miR-296-5p	-0.2397331	0.00506562
hsa-miR-519e*	-0.24763121	0.02028128
hsa-miR-184	-0.44333136	0.04380508

Table S2. miRNA profiling. Related to Figure 1 and Figure S1

miRNA profiling was performed on samples isolated from cells incubated at either 32°C or 37 °C. No miRNA expression had more than a 2-fold difference between cooled and control.

Decreased polysomal association

Top Networks

ID	Associated Network Functions	Score
1	Cell Death, Liver Damage, Lipid Metabolism	49
2	Nucleic Acid Metabolism, Small Molecule Biochemistry, Cellular Assembly and Organization	32
3	Connective Tissue Disorders, Dermatological Diseases and Conditions, Developmental Disorder	32
4	Reproductive System Development and Function, Tissue Morphology, Tissue Development	21
5	Infectious Disease, Molecular Transport, Renal and Urological System Development and Function	8

Molecular and Cellular Functions

Name	p-value	# Molecules
DNA Replication, Recombination, and Repair	3.18E-04 - 4.64E-02	6
Carbohydrate Metabolism	2.88E-03 - 3.50E-02	7
Cell Cycle	3.95E-03 - 4.64E-02	6
Cell Death	3.95E-03 - 4.92E-02	18
Cell-To-Cell Signaling and Interaction	3.95E-03 - 4.26E-02	6

Top Canonical Pathways

Name	p-value	Ratio
Induction of Apoptosis by HIV1	1.71E-03	3/66 (0.045)
Mitochondrial Dysfunction	1.56E-02	3/174 (0.017)
CD27 Signaling in Lymphocytes	1.73E-02	2/57 (0.035)
Oxidative Phosphorylation	1.89E-02	3/159 (0.019)
Ubiquinone Biosynthesis	3.12E-02	2/114 (0.018)

Table S3. Translational profiling. Related to Figure 1 and Figure S1

Functional classification of genes down-regulated at translational level following cold shock. Top significant networks, molecular functions and canonical pathways associated with the differentially expressed genes identified by Ingenuity Pathway Analysis.

"Slow" mRNAs, less polysomally associated	Accession	Cellular Component (GO terms)	Biological Process (GO terms)
**PLXND1**	NM_015103	GO:0005887 = integral component of plasma membrane	GO:0071526 = semaphorin-plexin signaling pathway
**ATP5F1**	NM_001688	GO:0000276 = mitochondrial proton-transporting ATP synthase complex<br>GO:0005743 = mitochondrial inner membrane	GO:0015986 = ATP synthesis coupled proton transport
**RECK**	NM_021111	GO:0005886 = plasma membrane<br>GO:0031225 = anchored component of membrane	GO:0030198 = extracellular matrix organization
**RTN3**	NM_001265589	GO:0000139 = Golgi membrane<br>GO:0005789 = ER membrane	GO:0006915 = apoptotic process<br>GO:0071786 = ER tubular network organization
**FOXN3**	NM_001085471	GO:0005634 = nucleus	GO:0006355 = regulation of transcription
**NDUFA9**	NM_005002	GO:0005743 = mitochondrial inner membrane	GO:0006120 = mitochondrial electron transport
**JTB**	NM_006694	GO:0005887 = integral component of plasma membrane	GO:0008637 = apoptotic mitochondrial changes
**CHPT1**	NM_020244	GO:0000139 = Golgi membrane	GO:0006656 = phosphatidylcholine biosynthetic process
**NOG**	NM_005450	GO:0005576 = extracellular region	GO:0001701 = in utero embryonic development
**CYP2E1**	NM_000773	GO:0000139 = Golgi membrane<br>GO:0031227 = intrinsic component of ER membrane	GO:0019373 = epoxygenase P450 pathway
**HSPA5/BIP**	NM_005347	GO:0005788 = ER lumen<br>GO:0034663 = ER chaperone complex<br>GO:0030176 = integral component of ER membrane	GO:0030968 = ER unfolded protein response
**KDM1A**	NM_001009999	GO:0005634 = nucleus<br>GO:0005667 = transcription factor complex	GO:0000122 = negative regulation of transcription
**GPX4**	NM_001039847	GO:0005739 = mitochondrion<br>GO:0005829 = cytosol	GO:0006749 = glutathione metabolic process
**CPB1**	NM_001871	GO:0005576 = extracellular region	GO:0006508 = proteolysis
**SLC25A5**	NM_001152	GO:0005743 = mitochondrial inner membrane<br>GO:0005887 = integral component of plasma membrane	GO:1901029 = negative regulation of mitochondrial outer membrane permeabilization involved in apoptotic signaling pathway
**HAVCR1**	NM_001099414	GO:0016021 = integral component of membrane	GO:0046718 = viral entry into host cell
**SIVA1**	NM_006427	GO:0005634 = nucleus<br>GO:0005739 = mitochondrion	GO:1901030 = positive regulation of mitochondrial outer membrane permeabilization involved in apoptotic signaling pathway
**RNF13**	NM_007282	GO:0000139 = Golgi membrane<br>GO:0005789 = ER membrane<br>GO:0016021 = integral component of membrane	GO:0051865 = protein autoubiquitination
**CCT6A**	NM_001762	GO:0005832 = chaperonin-containing T-complex	GO:0006457 = protein folding
**AATK**	NM_004920	GO:0005783 = ER<br>GO:0044295 = axonal growth cone<br>GO:0016021 = integral component of membrane	GO:0007420 = brain development<br>GO:0030517 = negative regulation of axon extension

Table S4. Related to Figure 1 and Figure S1 and 2

A list of mRNAs less polysomally associated following cooling and that contained poorly optimised codons. Interestingly, many of these proteins are membrane associated.

	Relative to Ala-IGC	
	Average	St Dev
Ala-IGC	1.00	0.0000
Ala-hGC	3.58	0.2675
Arg-ICG	5.10	1.3591
Arg-yCG	3.32	0.7445
Arg-CCT	2.19	0.6331
Arg-TCT	2.76	0.3822
Asn-GTT	6.45	1.7786
Asp-GTC	10.61	4.4094
Cys-GCA	11.42	3.9288
Gln-yTG	10.19	3.4363
Glu-yTC	12.41	5.1321
Glu-TTC	8.71	2.4103
Gly-sCC	8.32	1.5825
Gly-TCC	11.21	3.4035
His-GTG	3.96	0.4647
Ile-rAT	4.50	0.2619
Ile-TAT	1.92	0.0034
Leu-wAG	4.61	0.7049
Leu-CAG	7.42	0.5317
Leu-CAA	4.77	0.4465
Leu-TAA1	1.46	0.0800
Lys-CTT	8.36	3.0412
Lys-TTT1	7.69	2.1075
Met-i	10.76	2.8043
Met-e	7.48	0.7467
Phe-GAA	7.73	0.0323
Pro-hGG	5.03	1.4005
Sec-TCA2	0.76	0.1401
Ser-CGA	3.46	1.1038
Ser-wGA	3.38	0.4407
Ser-GCT	4.88	1.7870
Thr-mGT	7.28	1.3873
Thr-TGT1	4.09	0.7747
Thr-CGT2	1.04	0.2128
Trp-CCA	6.83	0.3551
Tyr-GTA	3.35	1.0032
Val-mAC	9.12	1.2182
Val-TAC	3.46	0.2984

Table S5. tRNA concentration. Related to Figure 1 and Figure S3

A table to show the relative tRNA expression in HEK-293 cells.

Gene Name	Average Speed on initial 20 codon stretch (codons/sec)
'PLXND1'	1.362297336
'ATP5F1'	1.442855541
'RECK'	1.510570878
'RPAP3'	1.521085566
'RTN3'	1.565637314
'FOXN3'	1.586957801
'NDUFA9'	1.601096139
'JTB'	1.615165029
'CHPT1'	1.615872734
'NOG'	1.625053918
'CYP2E1'	1.649410682
'HSPA5'	1.652127552
'KDM1A'	1.661677058
'GPX4'	1.676822335
'CPB1'	1.688679086
'SLC25A5'	1.707429543
'KCNJ13'	1.708290697
'HAVCR1'	1.7099814

Table S6. Decoding speed of first 20 amino acids. Related to Figure 1, Figure 2 and Figure S4

Calculation of the decoding speed of the first 20 codons of the mRNAs identified as associated with a decreased number of ribosomes following cooling. The table lists the top slowest 25%. This window of 20 codons was chosen because ribosome speed on the first codons following the start codon can be analysed without taking ribosome queuing effects into account, which would reduce the confidence of any predictions on speed properties. This analysis would identify a minimal sub-set of mRNAs where expression levels are controlled via the elongation stage, although it would also miss many such mRNAs where the speed-limiting sequence is located further downstream.

Supplemental Experimental Procedures

Antibodies

Antibodies were purchased from Sigma (β-Actin), Cell Signalling (Noggin, GBBR1, LDHA, eIF2α, eEF2), Abcam (RBM3 (human), RTN3), Santa Cruz (GAPDH, c-Myc), Proteintech (CIRP, RBM3 (mouse)) and D-GEN (ICSM35 for PrP).

Plasmids

The 5'UTR of *RTN3* was amplified by PCR from HEK293 cell cDNA using the following primer pairs:

RTN3 5’UTR fw   gactagGCATGCGCGCTCGCGC

RTN3 5’UTR rv   catgccatgGGCTACGCGAGCGAG

These sequences were cloned into pGL3 (Promega) upstream of luciferase firefly between *Spe*I and *Nco*I restriction enzyme sites and were verified by sequencing.

Transfection

SiRNAs (siGENOME Non-Targeting siRNA Pool #1 and SMARTpool: ON-TARGETplus RBM3 siRNA, Dharmacon) transfection were performed using DharmaFECT 1 (Dharmacon) transfection reagent at a final concentration of 100 nM according to manufacturer instructions, after 24h cells were split and transfected with luciferase constructs 24h later.

DNA transfections: These were performed using Lipofectamine 2000 (Invitrogen) following the manufacturer’s instructions and cells harvested 24 hours after transfection. Luciferase activity was measured using a dual luciferase assay system from Promega. Co-transfection with *Renilla* luciferase was used as an internal normalisation control.

Lentivirus: Mouse RBM3 isoform-2 (NM_001166410.1) overexpression was induced using the pLenti CAMKII (RBM3) Rsv (GFPBsd) plasmid [S1]. Mouse RTN3

(NM_053076.3) overexpression was induced with pLenti CAMKII (RBM3) Rsv (GFPBsd) plasmid (Gen Target Inc). The pLenti CAMKII (empty) Rsv (GFPBsd) plasmid was used as control.

RTN3 down regulation was achieved by using pLenti H1 shRNA (m RTN3) sequence #3 Rsv (GFPBsd). This plasmid contains the following shRNA-RTN3 sense, anti-sense and loop sequences (sequence #3:

5'TATGTTGGGATTGCCCGGGATcgagATCCCGGGCAATCCCAACATA 3'. The pLenty H1 shRNA (negative control) Rsv (GFPBsd) containing the sequence

5'-GTCTCCACGCGCAGTACATTTcgagAAATGTACTGCGCGTGGAGAC- 3'

was used as control. Viruses were used with a final titre of 0.6-1.5 x 108 TU. Prion infected mice were injected with lentiviruses at 2 w.p.i.

RT-PCR Primers

Mouse:

mRBM3 RT fw	ACTCTTCGTAGGAGGGCTCA
mRBM3 RT rev	CTCAGAGATAGGCCCAAAGC
mRTN3 RT fw	AGGCCTACTTGGATGTGGAC
mRTN3 RT rev	CAGCTTCAAGGAGTCAACCA

Human:

hACTIN RT fw	GTACCACTGGCATCGTGATGG
hACTIN RT rev	CCGCTCATTGCCAATGGTGAT
hGAPDH RT fw	GCACAGTCAAGGCCGAGAAT
hGAPDH RT rev	GCCTTCTCCATGGTGGTGAA
RBM3 h RT fw	GCTATGGGAGTGGCAGGTAT
RBM3 h RT rev	GTAGCGGTCATAACCACCCT

Protein synthesis rate determination

Cells were methionine starved for 30 minutes before addition of medium containing 40 μCi/ml Trans^{35}S -Label. After 30 minutes incubation, cells were harvested, washed and lysed in passive lysis buffer (Promega). Hippocampal slices were prepared. Slices were allowed to recover in normal artificial cerebrospinal fluid buffer while being

oxygenated either at 37°C or at 26°C for 1h, incubated with ^{35}S methionine label for 1h, then homogenized. Cells and slices lysates were centrifuged and protein precipitated from the supernatant with 10% trichloroacetic (TCA). Following washes with 70% IMS and 100% acetone, ^{35}S-methionine incorporation was determined by scintillation counting. Counts were normalised to total protein in the cell lysates as determined by Bradford assay (Bio-Rad).

Western Blotting

Cells and hippocampi lysed in protein lysis buffer (50 mM Tris, 150 mM NaCl, 1% Triton X-100, 1% Na deoxycholate, 0.1% SDS and 125 mM sucrose) supplemented with Phos-STOP and protease inhibitors (Roche), followed by centrifugation and quantification. PrP^{Sc} was detected after Proteinase K digestion. Lysates were subject to electrophoresis on SDS-polyacrylamide gels and the proteins transferred to PDVF or Nitrocellulose membranes (Bio-Rad). Blots were probed with the relevant antibodies and HRP-conjugated secondary antibodies, detected by ECL Prime western blotting detection reagent (GE Healthcare). Quantification was performed using ImageJ software.

Northern Blot analysis

RNA was separated by electrophoresis through a 1% formaldehyde-containing agarose gel and then transferred to Zeta-probe membrane (Bio-Rad) overnight in 20XSSC buffer and fixed by UV crosslinking. DNA hybridization probes were prepared from 50 ng DNA template and radiolabeled with αP^{32} CTP by random priming using Klenow enzyme (New England Biolabs). After overnight hybridisation with the radioactive probe in Church-Gilberts Solution (140 mM Na_2PO_4, 70 mM NaH_2PO_4, 7% SDS) at 55˚C, the membrane was washed twice with 2X SSC, 0.1% SDS, twice with 0.5X SSC, 0.1% SDS and twice with 0.1X SSC, 0.1% SDS.

Sucrose density gradient analysis

Cells were treated with cycloheximide at 100 µg/ml for 3 minutes prior to harvesting, pellets lysed in gradient buffer (300 mM NaCl, 15 mM MgCl2, 15 mM Tris pH 7.5 containing 1 mg/ml heparin sulphate and 100 µg/ml cycloheximide) plus 1% Triton X-100. The hippocampal tissues were dissected in ice-cold gradient buffer, and then homogenized in gradient buffer containing RNase inhibitors and 1.2% TritonX-100. Post nuclear supernatants were layered on 10-60% (w/v) sucrose gradients of gradient buffer. Gradients were centrifuged at 38,000 rpm for 2 hours at 4°C in a SW40Ti rotor (Beckman Coulter) then separated through a live optical density (OD) 254 nm UV spectrometer (Isco). Gradients were fractionated with continuous monitoring at 254 nm. Fractions of equal volume were collected directly into guandine-HCl, to give a final concentration of 5.8 M, and the RNA precipitated by addition of 1 volume of 100% ethanol. The RNA was resuspended in water and further purified by sodium acetate/ethanol precipitation.

Transcriptional Profiling

The human cDNA microarrays contained a set of total human cDNA clones. Total RNA from cells cultured either 24 hr at 37°C or at 32°C was extracted using TRIZOL (Invitrogen). Fluorescently labelled DNA probes were generated from equal proportions of RNAs (approximately 5µg) of 37°C RNA (Cy5) and 32°C RNA (Cy3) and hybridised to the arrays as described previously/ Microarray slides were scanned using a GenePix 4200B microarray scanner and GenePix Pro 6.0 software (Axon Instruments).

miRNA microarray

To investigate change in miRNA expression, miRNA microarray was carried out using a miRNA probe set from Exiqon (Exiqon, Vedbaek, Denmark) printed in-house.

RNA samples were labelled using FlashTagTM RNA labelling kit from Genisphere according to manufacturer instruction. Briefly 1.5ug of RNA was polyA tailed 15 min at 37°C followed by the labelling with a fluorophore (control with alexa 550 and coldshock with alexa-647) using T4 DNA ligase at room temperature for 30 min and then hybridised to the array at 52°C overnight. Following hybridisation slides were washed for 2 min in each wash solution (wash 1: 2X SSC, 0.2 %SDS; wash 2: 2X SSC; wash 3: 0.2X SSC), dried by centrifugation and scanned using a GenePix 4200B microarray scanner and GenePix Pro 6.0 software (Axon Instruments).

Analysis of Microarray Data

GenePix Pro 6.0 was used to quantify fluorescence intensities for individual spots on the microarray. All statistical analysis was performed in the statistical environment R, version 2.6.1 and the Limma package [S2]. Within-array normalisation using the print tip loess method and between-array normalisation using the scale method were performed on the data. Differentially expressed genes were then identified using the limFit and topTable functions. Genes which showed a fold change >2 and P-value < 0.05 were considered as potential candidates for altered distribution following coldshock at a statistically significant level. Once candidate genes were identified the sequence of the accession attached to each clone ID was search against the NCBI nucleotide database using nucleotide BLAST, with an E-value cut off of 0.001. The best match Refseq sequence(s) were chosen automatically for each clone ID. If no RefSeq hits were retrieved, non-RefSeq hits were checked manually for potential matches.

Immunoprecipitation reactions

Post-nuclear extracts were incubated with either anti-RBM3 antibody or IgG coated protein G magnetic beads which were washed (250 mM Tris pH 7.4, 300 mM NaCl, 5

mM $MgCl_2$, 0.5% NP40, 1 mM DTT and then resuspended in 200 µl buffer (50 mM Tris pH 7.4, 150 mM NaCl, 1 mM $MgCl_2$, 0.05% NP40) containing 0.1% SDS and 60 µg proteinase K. RNA was extracted using Trizol LS (Invitrogen) according to manufacturer instructions.

Supplemental References

S1. Peretti, D., Bastide, A., Radford, H., Verity, N., Molloy, C., Martin, M.G., Moreno, J.A., Steinert, J.R., Smith, T., Dinsdale, D., et al. (2015). RBM3 mediates structural plasticity and protective effects of cooling in neurodegeneration. Nature 518, 236-239.

S2. Smyth, G.K. (2004). Linear models and empirical bayes methods for assessing differential expression in microarray experiments. Stat Appl Genet Mol Biol *3*, Article3.
